# Design, synthesis, and *in silico* studies of new quinazolinones tagged thiophene, thienopyrimidine, and thienopyridine scaffolds as antiproliferative agents with potential p38α MAPK kinase inhibitory effects†

**DOI:** 10.1039/d4ra06744d

**Published:** 2025-01-16

**Authors:** Aisha A. Alsfouk, Ismail M. M. Othman, Manal M. Anwar, Asmaa Saleh, Eman S. Nossier

**Affiliations:** a Department of Pharmaceutical Sciences, College of Pharmacy, Princess Nourah bint Abdulrahman University P. O. Box 84428 Riyadh 11671 Saudi Arabia; b Department of Chemistry, Faculty of Science, Al-Azhar University Assiut 71524 Egypt; c Department of Therapeutic Chemistry, Pharmaceutical and Drug Industries Research Institute, National Research Centre El-Bohouth Street, Dokki, P. O. Box 12622 Cairo Egypt manal.hasan52@live.com; d Pharmaceutical Medicinal Chemistry and Drug Design Department, Faculty of Pharmacy (Girls), Al-Azhar University Cairo 11754 Egypt dr.emannossier@gmail.com dremannossier@azhar.edu.eg; e The National Committee of Drugs, Academy of Scientific Research and Technology Cairo 11516 Egypt

## Abstract

The current work focuses on the creation of novel derivatives of the quinazolinone ring system, with various substituted thiophene, thienopyrimidine, and thienopyridine scaffolds 3a,b–11. Employing the standard MTT assay, every target compound's *in vitro* antiproliferative efficacy was evaluated in comparison with doxorubicin against both normal WI-38 cells and various cancer cell lines. Derivatives 6, 8a, and 8b demonstrated the most potent activity, alongside their safety profiles against WI-38. The *in vitro* enzyme assay showed that the new analogues had a better ability to inhibit p38α MAPK kinase than SB 202190 (IC_50_s = 0.18 ± 0.02, 0.23 ± 0.05, 0.31 ± 0.04, and 0.27 ± 0.06 μM, respectively). Additionally, apoptosis tests conducted on MCF-7 cells revealed that 6, 8a, and 8b significantly increased the levels of Bax (by approximately 7.31, 13.8, and 8.86 fold) and caspase 3 (by approximately 3.55, 4.22, and 3.87 fold), respectively, in comparison to the untreated cells. They decreased the amount of Bcl-2 by ∼1.99, 3.69, and 2.66 fold, respectively. The most powerful counterpart, 8a, underwent additional investigation of the cell cycle and apoptosis. It caused necrotic and apoptotic effects in the late stages and stopped the MCF-7 cell cycle at the G2/M phase. Based on the molecular docking study, candidates 6, 8a, and 8b all fit well within p38α MAPK kinase, with energy scores of −10.88, −11.28, and −10.96 kcal mol^−1^, respectively. Based on the *in silico* computer examination of physico-chemical and ADMET properties, the latter analogues seem to be promising candidates for further development and optimization in research.

## Abstract 2

The current work focuses on the creation of novel derivatives of the quinazolinone ring system, with various substituted thiophene, thienopyrimidine, and thienopyridine scaffolds 3a,b–11.

## Graphical abstract

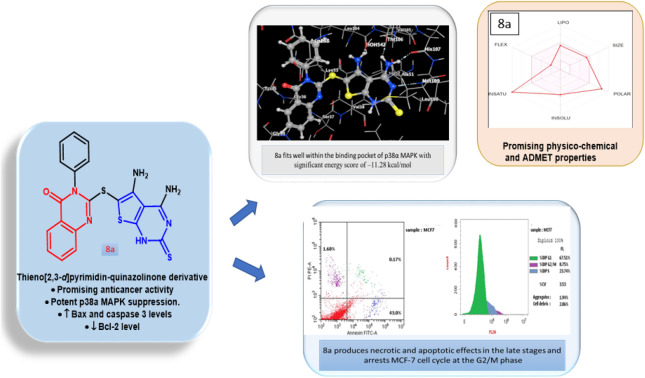

## 1. Introduction

Targeted therapy, which regulates cell development and targets specific proteins, is thought to be a pinpoint cancer treatment approach. A class of serine/threonine protein kinases called mitogen-activated protein kinases (MAPKs) are widely distributed in immunological, inflammatory, and endothelial cells.^1–4^ They are necessary for functions within cells, including cell division, death, and inflammation, as well as regulating the channels of signaling. The MAPK family is subdivided into four groups: p38 MAPKs, extracellular signal-regulated kinases (ERK1 and 2), and c-Jun N-terminal kinase 1 (JNK1).^5,6^ The main differences between these kinases, aside from their activation in response to distinct stimuli, are in the size and sequencing of their activation loops. p38 protein kinases in mammals are further divided into four isoforms (p38α, p38β, p38γ, and p38δ).^7^ Among them, p38α has been the most thoroughly researched isoform, which is essential for controlling pro-inflammatory signaling as well as the production and function of important pro-inflammatory cytokines, including interleukin-1 beta (IL-1β) and tumor necrosis factor alpha (TNF-α).^7^

Elevated p38α MAPK levels are associated with various cancer types, including thyroid, liver, breast, colon, and lung malignancies.^8,9^ There is ample evidence to suggest efficient function of excessively expressed p38α MAPK as a tumor booster, primarily through impairing control of cell cycle and promoting anti-apoptotic mechanisms.^10–13^ Due to its significant function, numerous cancer processes are linked to p38α MAPK, which is considered a significant therapeutic anticancer target. This has rendered the use of p38α-specific small-molecule inhibitors as pharmacologic anticancer therapies possible.^1,3,14^ Up to now, no FDA-approved medication for marketing has been developed using selective p38α inhibitors, despite tremendous efforts in their design and synthesis.^5^ Nonetheless, at various phases of assessment, a limited quantity of p38α MAPK suppressors have advanced to clinical development and research.

**Fig. 1:**
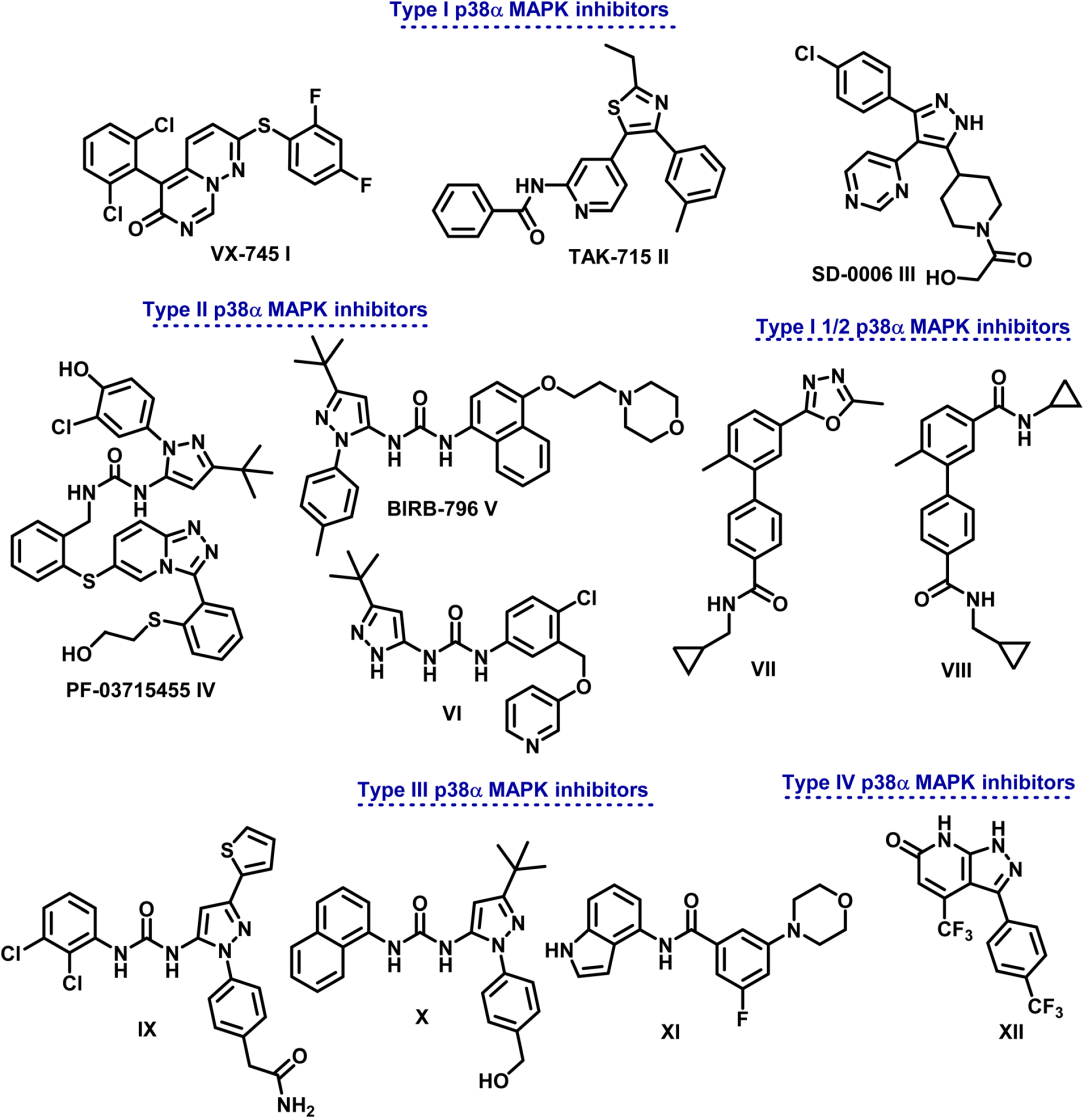
Different types of p38α MAPK inhibitors.

Based on the distinctive manner of binding within the kinase active site, p38α MAPK inhibitors are divided into different categories (Fig. 1). In a DFG-in conformation, type I inhibitors bind at the point where the ATP adenine ring occurs. Through hydrogen bonding, they engage with p38α MAPK to link the Met109 acid that is in connection with the hydrophobic area I.^5^

Different types of pyrimido[1,6-*b*]pyridazine I, pyridinyl II, and pyrimidinyl III derivatives are class I selective ATP-competitive p38α MAPK suppressors that have been tested in both I and II phases of clinical research.^15–17^

Additionally, class II P38α MAPK inhibitors like IV (PF-03715455), V (BlRB-796), and VI bind in a DFG-out conformation to the hydrophobic pocket close to the ATP binding site. Phase II clinical studies are underway.^18^ Adhering to an allosteric site allows them to establish more hydrogen bonds with the hinge Met109 residue. On the other hand, type III inhibitors bind to an allosteric pocket next to the ATP-binding pocket.^19,20^

Because they are non-competitive ATP, they can attach themselves directly to the dormant protein. Molecules IX–XI serve as examples.^21^ In order to generate conformational changes that render a protein inactive, type IV inhibitors—ATP non-competitive suppressors—connect with the allosteric site that is separate from the ATP-binding region. One example of this class of inhibitors is compound XII (Fig. 1).^22^

Research has demonstrated the distinct pharmacophores of drugs that inhibit p38α MAPK, with heterocyclic scaffolds such as imidazole, thiazole, oxadiazole, pyridine, and pyrimidine serving as intriguing cores that can be fused or connected to other heterocyclic rings.^23–26^

In medicinal chemistry, heterocyclic scaffolds are regarded as important molecular structures. They generate a wide range of biological actions aside from being found in the framework of several biological components, including hemoglobin, hormones, vitamins, RNA, DNA, and heterocyclic compounds.^27,28^ As a result, heterocyclic molecules have long been desirable synthetic targets in drug design and discovery owing to their biological significance and structural diversity.

**Fig. 2:**
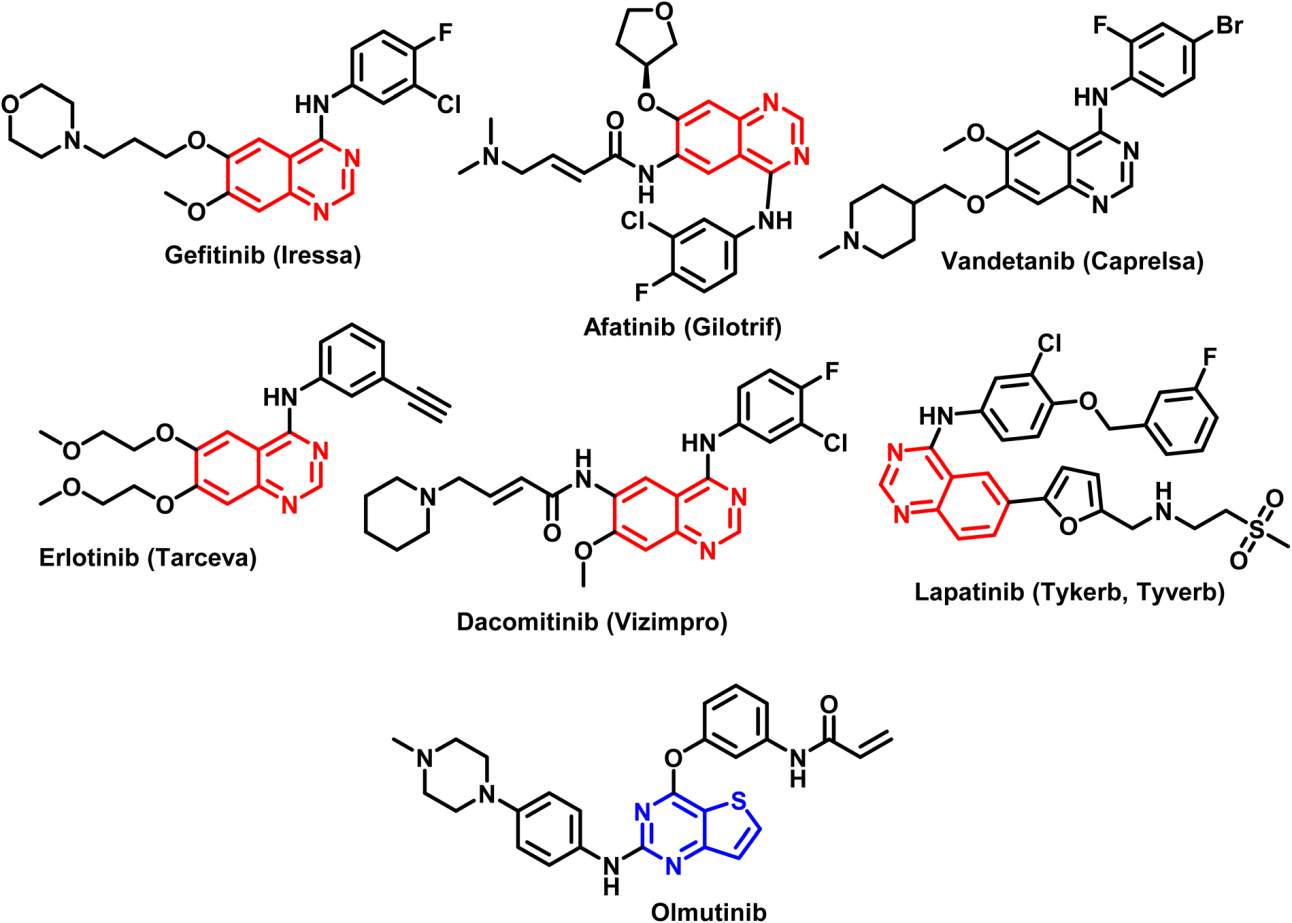
Different FDA approved drugs based on quinazoline and thienopyrimidine ring systems.

The pharmacophore quinazoline is a class of heterocyclic compounds that has recently developed into a remarkably versatile framework due to its many pharmacological characteristics, which include antiviral,^29^ antibacterial,^30^ anti-inflammatory,^31,32^ antihypertensive,^33^ antioxidant,^34^ and anticancer.^35^ Certain cancer chemotherapeutic drugs, such as quinazoline and its derivatives, have proven to be highly effective in treating solid tumors. The FDA has approved quinazoline-based anticancer medications as Gefitinib, Afatinib, Vendetanib, Erlotinib, Lapatinib, and Dacomitinib (Fig. 2).^36^ Furthermore, thieno[2,3-*b*]pyrimidines and thieno[2,3-*b*]pyridines constitute other two classes of heterocyclic molecules with therapeutic potential for a wide range of illnesses. Several substituted thieno[2,3-*b*]pyrimidines and thienopyridine compounds have been identified as potent antiproliferative agents against a variety of human cancer cell lines in recent studies.^37–40^ According to several investigations, hydrogen bonds between the heteroatoms of the molecules and the backbone NH group of various amino acid residues of the target enzyme are the primary mechanism through which heterocyclic rings interact.^41–43^ Olmutinib (Fig. 2) is an EGFR tyrosine kinase inhibitor that was recently authorized by the FDA to treat breast and non-small cell lung cancers.^44^

Major fundamental barriers to successful clinical use of these drugs have persisted, including the high toxicity of various commonly used chemotherapeutics, the insensitivity of different tumor cells to anticancer drugs, and the incapacity of some antitumor medications to exert their effects in specific cases.

High toxicity of many frequently utilized chemotherapy remedies, inability of some antitumor medications to achieve the desired effect in particular cases, as well as insensitivity of various malignant cells to anticancer drugs are among the major fundamental obstacles that have persisted to the successful clinical use of these drugs.^45,46^

To enhance patient quality of life and overall survival, it is crucial to find new chemotherapeutics with clearly distinguished mechanisms of action that can be used individually or in combination. Integrating more than one scaffold into a single molecule also provides a workable hybrid pharmacophore strategy for brand-new anticancer prospects.^47–49^

**Fig. 3:**
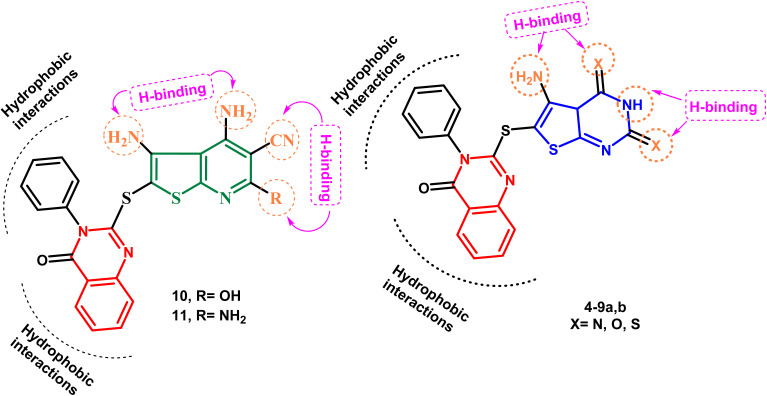
New thienopyrimidine and thienopyridine derivatives: postulated hypothesis models.

Keeping these factors in mind and also depending upon the effect of variations in ring size, molecular orientation, and the total number of heteroatoms that could contribute additional hydrogen binding with the active ATP binding sites, the goal of the current work was to generate novel molecules based on quinazoline, thienopyrimidine, and thienopyridine rings coupled with various heterocyclic cores with documented anticancer potential, aiming to establish new anticancer agents with potential p38α MAP kinase inhibitory action. Fig. 3 illustrates the proposed layout of the new derivatives.

## 2. Experimental

### 2.1. Chemistry

The instruments used for measuring the melting points, spectral data (IR, mass, ^1^H NMR and ^13^C NMR) and elemental analysis are provided in details in ESI material.†

The ESI material† contains a detailed description of the instruments used to measure the melting points, elemental analyses and spectrum data (^1^H NMR, ^13^C NMR, IR and mass).

The starting compound 2-mercapto-3-phenylquinazolin-4-one was prepared according to the reported method.^50^

#### 2.1.1. Synthesis of 2-((4-oxo-3-phenyl-3,4-dihydroquinazolin-2-yl)thio)acetonitrile (1)

After 30 minutes of stirring an equimolar combination of sodium acetate (10 mmol) and 2-mercapto-3-phenylquinazolin-4-one (10 mmol) in dry DMF (20 mL), chloroacetonitrile (10 mmol) was added. After 5 h of reflux, the reaction mixture was allowed to settle to room temperature before being added to ice-cold water. To obtain a pure product 1 as white crystals, the precipitate was filtered out and recrystallized from ethanol.

Yield 86%; mp 119–121 °C; IR (*ν*_max_/cm^−1^): 3034 (CH-rom.), 2925 (CH-aliph.), 2215 (CN), 1659 (CO); ^1^H NMR (400 MHz, DMSO-*d*_6_): *δ* 4.20 (s, 2H, CH_2_), 7.27–7.98 (m, 9H, Ar-H); ^13^C NMR (101 MHz, DMSO-*d*_6_): 33.7 (CH_2_), 115.2 (CN), 120.1, 126.7, 126.8, 126.9, 129.9, 130.0, 130.8, 135.2, 147.1, 156.5, 165.6 (CO). Anal. calcd for C_16_H_11_N_3_OS (293.34): C, 65.51; H, 3.78; N, 14.32; S, 10.93%. Found: C, 65.73; H, 3.59; N, 14.54; S, 10.71%.

#### 2.1.2. General procedure for the synthesis of thiophene derivatives 3a,b

Active methylene reagents, such as malononitrile or ethyl cyanoacetate (10 mmol), were added to a mixture of phenylquinazolin-2-yl thioacetonitrile derivative 1 (10 mmol) and elemental sulfur (10 mmol) in absolute ethanol (20 mL) with a few drops of triethylamine as a catalyst. In each case, the reaction mixture was heated under reflux for seven hours before being poured into a mixture of ice and water with a few drops of HCl to cool it down and neutralize it. In each case, the resultant solid product was gathered through filtering and crystallized from dioxane to yield structures 3a,b.

##### 2.1.2.1. 2,4-Diamino-5-((4-oxo-3-phenyl-3,4-dihydroquinazolin-2-yl)thio)thiophene-3-carbonitrile (3a)

Yield 88%, brown crystal; mp 197–199 °C; IR (*ν*_max_/cm^−1^): 3223, 3190 (2NH_2_), 3043 (CH-arom.), 2218 (CN), 1673 (CO); ^1^H NMR (400 MHz, DMSO-*d*_6_) *δ*: 6.14 (s, 2H, NH_2_, D_2_O exchangeable), 6.61 (s, 2H, NH_2_, D_2_O exchangeable), 7.44–8.12 (m, 9H, Ar-H); ^13^C NMR (101 MHz, DMSO-*d*_6_): 110.6, 115.1 (CN), 120.4, 121.8, 122.2, 122.5, 124.3, 127.1, 127.3, 129.5, 129.6, 130.4, 134.2, 141.4, 150.9, 151.5, 166.2 (CO). Anal. calcd for C_19_H_13_N_5_OS_2_ (391.47): C, 58.29; H, 3.35; N, 17.89; S, 16.38%. Found: C, 58.51; H, 3.57; N, 17.66; S, 16.59%.

##### 2.1.2.2. Ethyl 2,4-diamino-5-((4-oxo-3-phenyl-3,4-dihydroquinazolin-2-yl)thio)thiophene-3-carboxylate (3b)

Yield 84%, brown crystal; mp 193–195 °C; IR (*ν*_max_/cm^−1^): 3211, 3165 (2NH_2_), 3057 (CH-arom.), 1725, 1686 (2CO); ^1^H NMR (400 MHz, DMSO-*d*_6_) *δ*: 1.21 (t, 3H, *J* = 7.2 Hz, CH̲_2_CH_3_), 4.12 (q, 2H, *J* = 7.2 Hz, OCH̲_2_CH_3_), 6.31 (s, 2H, NH_2_, D_2_O exchangeable), 6.84 (s, 2H, NH_2_, D_2_O exchangeable), 7.48–8.09 (m, 9H, Ar-H); ^13^C NMR (101 MHz, DMSO-*d*_6_): 14.5, 61.3, 120.1, 122.7, 125.6, 125.8, 126.6, 126.9, 129.8, 30.0, 130.5, 131.5, 134.7, 135.2, 148.7, 156.6, 161.1, 163.3, 166.1. Anal. calcd for C_21_H_18_N_4_O_3_S_2_ (438.52): C, 57.52; H, 4.14; N, 12.78; S, 14.62%. Found: C, 57.75; H, 4.36; N, 12.54; S, 14.83%.

#### 2.1.3. General procedure for the synthesis of thienopyrimidines 4 and 5

A solution of aminothiophene-3-carbonitrile derivative 3a (10 mmol) in different reagents, namely, acetic anhydride and/or formic acid (20 mL), was heated under reflux for 24 h. Compounds 4 and 5 were generated by filtering and recrystallizing the precipitated material from methanol after the reaction solution had cooled and been added to ice water while being stirred.

##### 2.1.3.1. 5-Amino-2-methyl-6-((4-oxo-3-phenyl-3,4-dihydroquinazolin-2-yl)thio)thieno[2,3-*d*]pyrimidin-4(3*H*)-one (4)

Yield 67%, buff crystals; mp 278–280 °C; IR (*ν*_max_/cm^−1^): 3352, 2277 (NH_2_), 2201 (NH), 3064 (CH-arom.), 2985 (CH-aliph.), 1697, 1683 (2CO); ^1^H NMR (400 MHz, DMSO-*d*_6_) *δ*: 2.13 (s, 3H, CH_3_), 6.71 (s, 2H, NH_2_, D_2_O exchangeable), 7.06–8.46 (m, 10H, Ar-H + NH); ^13^C NMR (101 MHz, DMSO-*d*_6_): 21.3, 121.1, 125.2, 126.1, 126.6, 126.9, 127.5, 128.8, 129.9, 130.0, 130.1, 130.6, 132.1, 147.6, 148.2, 156.3, 158.1, 162.9, 167.6. Anal. calcd for C_21_H_15_N_5_O_2_S_2_ (433.51): C, 58.18; H, 3.49; N, 16.16; S, 14.79%. Found: C, 58.39; H, 3.71; N, 16.37; S, 14.58%.

##### 2.1.3.2. 5-Amino-6-((4-oxo-3-phenyl-3,4-dihydroquinazolin-2-yl)thio)thieno[2,3-*d*]pyrimid in-4(3*H*)-one (5)

Yield 65%, pale brown crystals; mp 273–275 °C; IR (*ν*_max_/cm^−1^): 3344, 2269 (NH_2_), 2175 (NH), 3045 (CH-arom.), 1695, 1667 (2CO); ^1^H NMR (400 MHz, DMSO-*d*_6_) *δ*: 6.18 (s, 2H, NH_2_, D_2_O exchangeable), 6.87–8.75 (m, 10H, Ar-H + CH-pyrimidine), 8.83 (s, 1H, NH, D_2_O exchangeable); ^13^C NMR (101 MHz, DMSO-*d*_6_): 122.2, 124.3, 124.5, 125.4, 126.8, 127.9, 129.1, 130.0, 130.7, 131.0, 131.1, 138.8, 146.6, 148.2, 158.8, 159.0, 164.7, 166.2. Anal. calcd for C_20_H_13_N_5_O_2_S_2_ (419.48): C, 57.26; H, 3.12; N, 16.70; S, 15.29%. Found: C, 57.47; H, 3.33; N, 16.52; S, 15.49%.

#### 2.1.4. 2-((4,5-Diaminothieno[2,3-*d*]pyrimidin-6-yl)thio)-3-phenylquinazolin-4(3*H*)-one (6)

For a duration of 12 hours, equimolar volumes of formamide (20 mL) and aminothiophene-3-carbonitrile derivative 3a (10 mmol) were heated under reflux. After cooling, the reaction mixture was added to 30 milliliters of ice-cold water. After filtering and crystallizing the resultant solid from dioxane, product 6 was obtained as green crystals; yield 76%; mp 287–289 °C; IR (*ν*_max_/cm^−1^): 3349, 2318, 3223, 3157 (2NH_2_), 3072 (CH-rom.), 1656 (CO); ^1^H NMR (400 MHz, DMSO-*d*_6_) *δ*: 6.25 (s, 2H, NH_2_, D_2_O exchangeable), 7.43–8.11 (m, 11H, Ar-H + NH_2_), 8.65 (s, 1H, CH-pyrimidine); ^13^C NMR (101 MHz, DMSO-*d*_6_): 121.0, 125.2, 126.5, 126.9, 127.2, 127.4, 127.9, 129.8, 130.1, 130.5, 135.2, 135.4, 147.5, 148.9, 150.7, 156.7, 159.1, 163.3. Anal. calcd for C_20_H_14_N_6_OS_2_ (418.49): C, 57.40; H, 3.37; N, 20.08; S, 15.32%. Found: C, 57.62; H, 3.59; N, 20.30; S, 15.53%.

#### 2.1.5. 2-((5-Amino-2,4-dithioxo-1,2,3,4-tetrahydrothieno[2,3-*d*]pyrimidin-6-yl)thio)-3-phenylquinazolin-4(3*H*)-one (7)

Compound 3a (10 mmol) and carbon disulfide (15 mmol) combined with pyridine (20 mL) were refluxed for 10 hours in a water bath. The reaction mixture was cooled to room temperature, added to ice-cold water, and neutralized with diluted HCl once the reaction was complete (TLC). To get compound 7 as dark brown crystals, the precipitated product was filtered out, washed with water, dried, and then recrystallized from EtOH/dioxane; yield 66%; mp 265–267 °C; IR (*ν*_max_/cm^−1^): 3336, 32 251 (NH_2_), 3183, 3112 (2NH), 3050 (CH-rom.), 1682 (CO); ^1^H NMR (400 MHz, DMSO-*d*_6_) *δ*: 6.20 (s, 2H, NH_2_, D_2_O exchangeable), 7.12–8.15 (m, 10H, Ar-H + NH), 12.50 (s, 1H, NH, D_2_O exchangeable); ^13^C NMR (101 MHz, DMSO-*d*_6_): 119.6, 120.1, 124.5, 126.0, 126.4, 126.7, 126.8, 129.9, 130.1, 130.5, 136.1, 136.6, 148.6, 151.1, 158.5, 164.3, 182.4. Anal. calcd for C_20_H_13_N_5_OS_4_ (467.61): C, 51.37; H, 2.80; N, 14.98; S, 27.43%. Found: C, 51.58; H, 2.60; N, 14.77; S, 27.65%.

#### 2.1.6. General procedure for the synthesis of compounds 8a,b

Compound 3a (10 mmol) combined with either urea (10 mmol) or thiourea (10 mmol) in 30 mL of ethanolic sodium ethoxide. In each case, the reaction mixture was heated under reflux for nine hours, cooled, and then put onto crushed ice before being neutralized with diluted hydrochloric acid. In order to produce structures 8a,b, the resulting solid product in each case was recovered by filtration and recrystallized from EtOH/dioxane.

##### 2.1.6.1. 2-((4,5-Diamino-2-thioxo-1,2-dihydrothieno[2,3-*d*]pyrimidin-6-yl)thio)-3-phenyl quinazolin-4(3*H*)-one (8a)

Yield 72%, yellow crystals; mp 292–294 °C; IR (*ν*_max_/cm^−1^): 3405, 3315 (2NH_2_), 2210 (NH), 3082 (CH-arom.), 1675 (CO); ^1^H NMR (400 MHz, DMSO-*d*_6_) *δ*: 6.28 (s, 2H, NH_2_, D_2_O exchangeable), 6.86–8.72 (m, 9H, Ar-H), 8.35 (s, 1H, NH_2_, D_2_O exchangeable), 8.81 (s, 1H, NH, D_2_O exchangeable); ^13^C NMR (101 MHz, DMSO-*d*_6_): 120.2, 120.6, 123.5, 126.4, 126.9, 127.6, 128.7, 129.3, 129.9, 130.0, 130.4, 130.9, 147.1, 152.3, 152.8, 156.5, 162.9, 181.1; MS: *m*/*z* = 450 [M^+^], 198 (100%). Anal. calcd for C_20_H_14_N_6_OS_3_ (450.56): C, 53.31; H, 3.13; N, 18.65; S, 21.35%. Found: C, 53.53; H, 3.35; N, 18.87; S, 21.56%.

##### 2.1.6.2. 4,5-Diamino-6-((4-oxo-3-phenyl-3,4-dihydroquinazolin-2-yl)thio)thieno[2,3-*d*]pyrimidin-2(1*H*)-one (8b)

Yield 70%, yellow crystals; mp 298–300 °C; IR (*ν*_max_/cm^−1^): 3392, 3278 (2NH_2_), 2196 (NH), 3059 (CH-arom.), 1689, 1664 (2CO); ^1^H NMR (400 MHz, DMSO-*d*_6_) *δ*: 6.17 (s, 2H, NH_2_, D_2_O exchangeable), 7.38–8.04 (m, 11H, Ar-H + NH_2_), 9.44 (s, 1H, NH, D_2_O exchangeable); ^13^C NMR (101 MHz, DMSO-*d*_6_): 110.5, 119.1, 120.0, 124.2, 126.5, 126.9, 127.2, 129.9, 130.0, 130.5, 134.1, 136.2, 147.6, 147.8, 155.2, 156.5, 163.6, 166.1; MS: *m*/*z* = 434 [M^+^], 182 (100%). Anal. calcd for C_20_H_14_N_6_O_2_S_2_ (434.49): C, 55.29; H, 3.25; N, 19.34; S, 14.76%. Found: C, 55.51; H, 3.46; N, 19.55; S, 14.54%.

#### 2.1.7. General procedure for the synthesis of thienopyridines 9–11

Reflux was employed to heat a solution of aminothiophene-3-carbonitrile 3a (10 mmol) and each of ethyl cyanoacetate, malononitrile, or ethyl acetoacetate (10 mmol) in ethyl alcohol (30 mL) containing 0.5 mL of triethylamine as a catalyst for nine hours. After cooling, the reaction mixture was added to water that had been chilled with ice, and it was acidified using diluted HCl. After filtering and recrystallizing the resultant solid product from EtOH and dioxane, the target compounds 10–12 were obtained, respectively.

##### 2.1.7.1. 3,4-Diamino-6-hydroxy-2-((4-oxo-3-phenyl-3,4-dihydroquinazolin-2-yl)thio)thieno[2,3-*b*]pyridine-5-carbonitrile (9)

Yield 68%, deep yellow crystals; mp 255–257 °C; IR (*ν*_max_/cm^−1^): 3399 (OH), 3246, 3137 (2NH_2_), 3035 (CH-arom.), 2221 (CN), 1698 (CO); ^1^H NMR (400 MHz, DMSO-*d*_6_) *δ*: 6.04 (s, 2H, NH_2_, D_2_O exchangeable), 6.16 (s, 2H, NH_2_, D_2_O exchangeable), 7.11–8.18 (m, 9H, Ar-H), 11.45 (s, 1H, OH, D_2_O exchangeable); ^13^C NMR (101 MHz, DMSO-*d*_6_): 109.6, 115.3, 121.1, 121.4, 123.9, 126.1, 126.6, 126.8, 126.9, 129.7, 130.0, 136.1, 136.1, 147.2, 148.6, 151.3, 157.5, 161.1, 163.7. Anal. calcd for C_22_H_14_N_6_O_2_S_2_ (458.52): C, 57.63; H, 3.08; N, 18.33; S, 13.99%. Found: C, 57.85; H, 3.29; N, 18.56; S, 13.77%.

##### 2.1.7.2. 3,4,6-Triamino-2-((4-oxo-3-phenyl-3,4-dihydroquinazolin-2-yl)thio)thieno[2,3*-b*]pyridine-5-carbonitrile (10)

Yield 74%, deep yellow crystals; mp 281–283 °C; IR (*ν*_max_/cm^−1^): 3267, 3220 (NH_2_), 3055 (CH-arom.), 2216 (CN), 1672 (CO); ^1^H NMR (400 MHz, DMSO-*d*_6_) *δ*: 6.43 (s, 2H, NH_2_, D_2_O exchangeable), 6.83, 6.85 (s, 4H, 2NH_2_, D_2_O exchangeable), 7.46–8.10 (m, 9H, Ar-H); ^13^C NMR (101 MHz, DMSO-*d*_6_): 108.5, 115.7, 120.0, 120.6, 121.4, 121.8, 122.5, 123.0, 127.3, 128.1, 129.7, 129.8, 131.6, 132.0, 140.3, 142.0, 156.4, 157.3, 160.8, 16.15. Anal. calcd for C_22_H_14_N_6_O_2_S_2_ (457.53): C, 57.75; H, 3.30; N, 21.43; S, 14.02%. Found: C, 57.53; H, 3.51; N, 21.64; S, 14.23%.

##### 2.1.7.3. 2-((5-Acetyl-3,4-diamino-6-hydroxythieno[2,3-*b*]pyridin-2-yl)thio)-3-phenyl quinazolin-4(3*H*)-one (11)

Yield 71%, deep yellow crystals; mp over 300 °C; IR (*ν*_max_/cm^−1^): 3492 (OH), 3351, 3312 (2NH_2_), 3078 (CH-arom.), 2963 (CH-aliph.), 1716, 1659 (2CO); ^1^H NMR (400 MHz, DMSO-*d*_6_) *δ*: 2.20 (CH_3_), 6.15 (s, 2H, NH_2_, D_2_O exchangeable), 6.58 (s, 2H, NH_2_, D_2_O exchangeable), 7.21–8.30 (m, 9H, Ar-H), 11.23 (s, 1H, OH, D_2_O exchangeable); ^13^C NMR (101 MHz, DMSO-*d*_6_): 22.2, 110.8, 119.3, 121.4, 121.9, 122.6, 124.0, 124.3, 127.7, 128.2, 129.3, 129.8, 137.9, 139.0, 147.6, 149.3, 153.6, 157.3, 158.4, 163.2, 186.4. Anal. calcd for C_23_H_17_N_5_O_3_S_2_ (475.54): C, 58.09; H, 3.60; N, 14.73; S, 13.49%. Found: C, 58.31; H, 3.82; N, 14.51; S, 13.72%.

### 2.2. Biological activity

#### 2.2.1. *In vitro* anticancer assessment

The MTT test was utilized for the screening process. Additional information was given in the ESI material.†

#### 2.2.2. *In vitro* p38α MAPK assay

The recombinant protein kinase utilized in the compound profiling process was cloned, generated, and purified using exclusive methods. Using an ADP-GloTM assay kit, stock solutions of the test compounds were produced and subsequently tested at a single concentration of 10 μM to determine their capacity to inhibit p38αMAPK. ESI† content included more information.

#### 2.2.3. Bax, Bcl-2, and caspase-3 assays

The quantity of human active caspase-3 was determined using the Invitrogen human active caspase-3 “Catalog # KHO1091” (Invitrogen Corporation, Camarillo, CA 93012, United States) ELISA kit. There are more specifics in the ESI data.†

#### 2.2.4. Cell cycle analysis

The most effective substance, 8a, was applied to the MCF-7 cell line for 24 hours at its IC_50_ concentration. ESI† content offered more information.

#### 2.2.5. Apoptosis assay

The BD Biosciences Annexin V-FITC apoptosis detection kit was utilized to measure the percentage of cells passing through apoptosis and identify the types of cell death, which include necrosis and apoptosis, depending on whether active compound 8a was present or absent. The procedure provided by the manufacturer was followed when conducting the experiment. ESI† content addressed more information.

## 3. Results and discussion

### 3.1. Chemistry

**Scheme 1:**
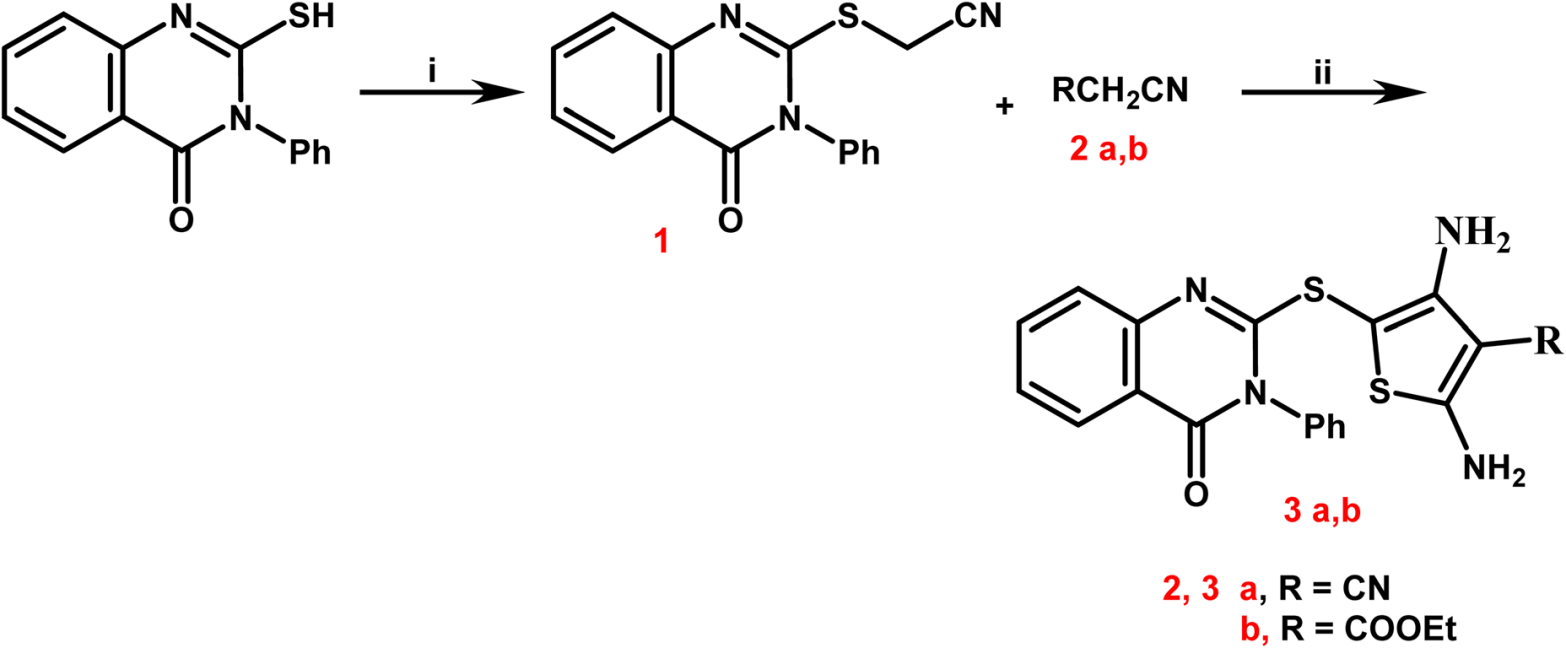
Synthetic path way for synthesis of 2,4-diamino-thiophene-3-carbonitriles 3a,b. Reagents and conditions: (i) chloro-acetonitrile, sodium acetate, dry DMF, stirring for 30 minutes, refluxing for 5 h; (ii) S, malononitrile and/or ethyl cyanoacetate, absolute ethanol, few drops of triethylamine, reflux for 7 h.

**Scheme 2:**
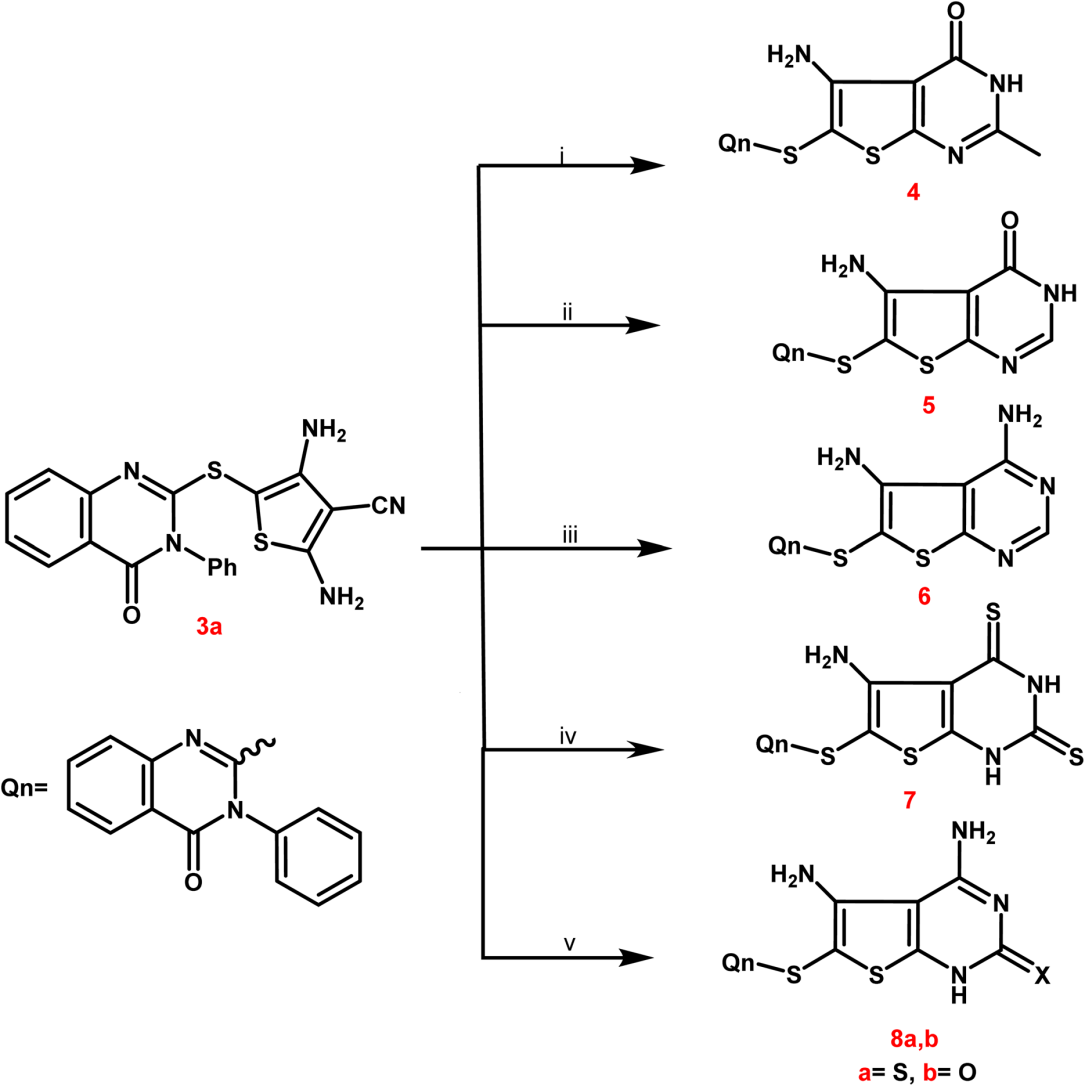
Synthetic path way for synthesis of different substituted thienopyrimidines 4–8a,b. Reagents and conditions: (i and ii) acetic anhydride and/or formic acid, reflux for 24 h; (iii) formamide, reflux for 12 h; (iv) carbon disulphide, pyridine, reflux on water bath for 10 h; (v) thiourea and/or urea, ethanolic sodium ethoxide, reflux for 9 h.

**Scheme 3:**
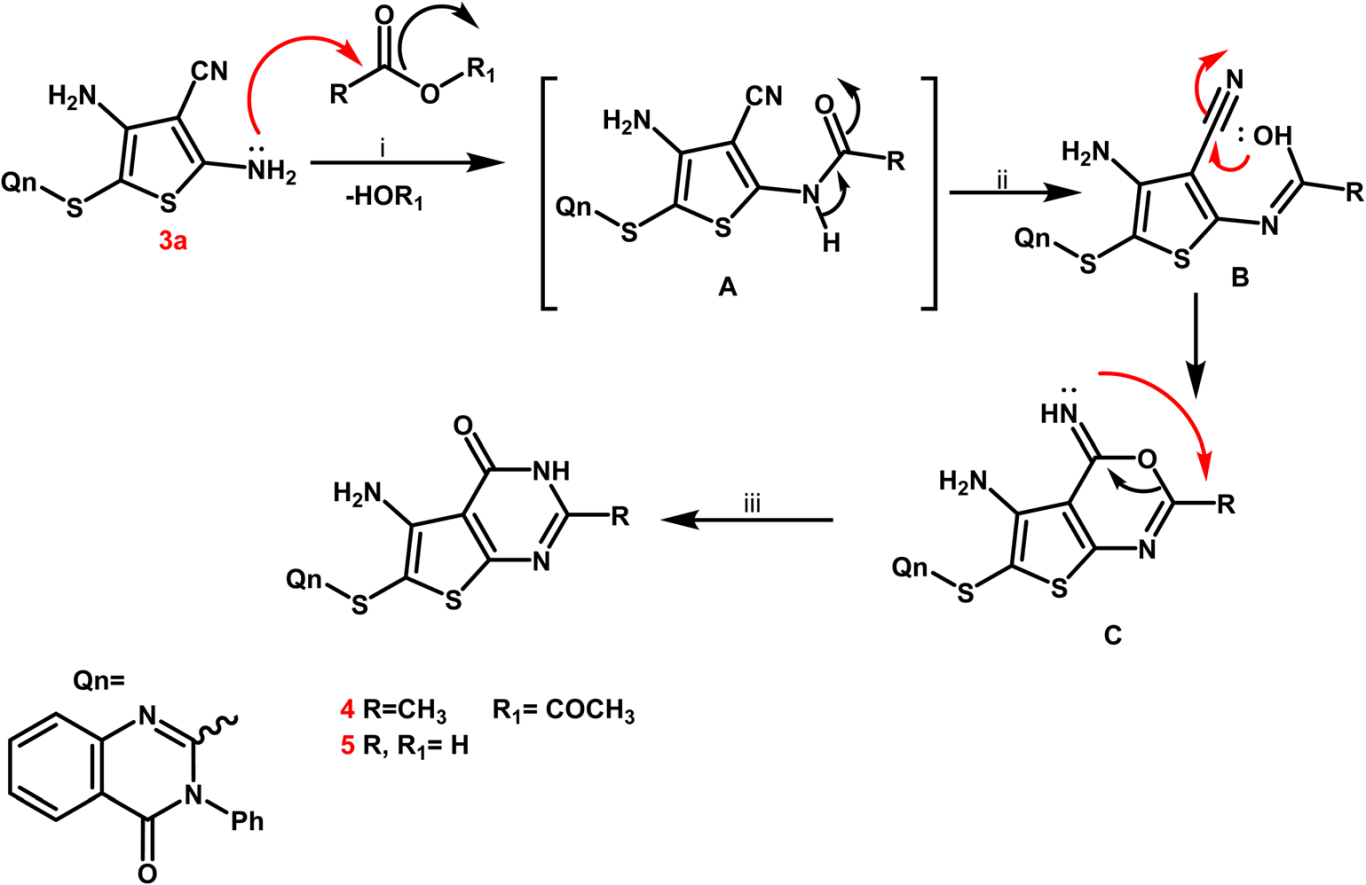
Mechanistic perspective for the synthesis of thienopyrimidine derivatives 4, 5.

**Scheme 4:**
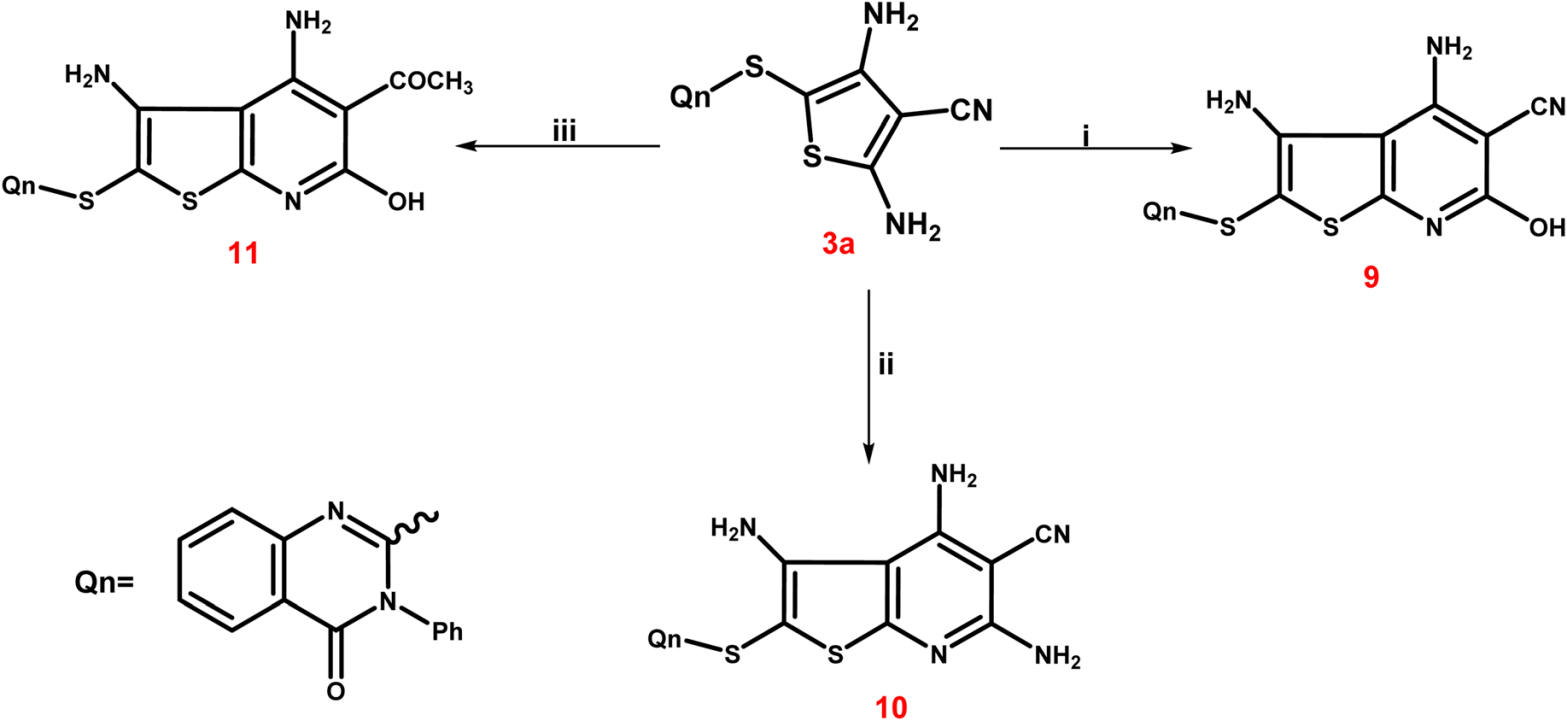
A synthetic route for creation of various substituted thienopyridines 9–11. Reagents and conditions; (i) ethyl cyanoacetate, ethanol, triethylamine, reflux for 9 h; (ii) malononitril, ethanol, triethylamine, reflux for 9 h; (iii) ethyl acetoacetate, ethanol, triethylamine, reflux for 9 h.

Newly targets 3a,b, 4–9a,b, and 10–12 were created as summarized in Schemes 1–4. Firstly, in Scheme 1, the key stating compound 2-mercapto-3-phenylquinazolin-4-one synthesized according to the documented method^50^ was permitted to react with chloroacetonitrile in dry DMF containing sodium acetate to generate the corresponding acetonitrile compound 1.

The 2,4-diamino-thiophene-3-carbonitriles 3a,b were obtained *via* the Gewald reaction of 1 in absolute ethanol containing a catalytic amount of triethylamine with sulphur and various active methylene reagents, namely malononitrile or ethyl cyanoacetate. Characteristic bands appeared at 3223–3165 cm^−1^ for 2NH_2_, 2218 cm^−1^ for CN, and 1725–1686 cm^−1^ for CO groups, according to the IR spectra of 3a,b. ^1^H NMR spectra of compounds 3a,b exhibited two D_2_O singlet signals at *δ* 6.13–6.84 ppm referring to 2NH_2_ groups, multiplet signals in the region 7.44–8.12 corresponding to the aromatic protons. Regarding to the carboxylate protons of 3b appeared as triplet–quartet signals at *δ* 1.21 and 4.12 ppm and at *δ* 14.53 and 61.37 ppm in its ^13^C NMR spectrum.

In Scheme 2, compound 3a was selected as a crucial antecedent to synthesis new targets 4–8a,b. Upon treatment of 3a different reagents, namely, acetic anhydride and/or formic acid, under reflux for 24 hours furnished the corresponding thieno[2,3-*d*]pyrimidin-4(3*H*)-one 4 and 5, respectively. Also, the condensation of 3a with an equimolar amount of formamide under reflux resulted in the creation of the corresponding 4,5-diaminothieno[2,3-*d*]pyrimidine analogue 6.

IR spectra of derivatives 4 and 5 revealed two distinctive bands at 1697–1667 cm^−1^ assignable to CO groups. Additionally, bands of NH and NH_2_ groups appeared at 3352–2201 cm^−1^, and the 2NH_2_ groups of compound 6 were represented by two forked bands at 3349–3157 cm^−1^.

Compounds 4 and 5's ^1^H NMR spectra showed multiplet signals at *δ* 6.87–8.75 ppm caused by aromatic protons, as well as D_2_O exchangeable singlets at *δ* 6.71–6.18 ppm due to NH_2_ groups. Compound 4 showed a singlet signal for its 2-CH_3_ protons at *δ* 2.13 ppm and at *δ* 21.34 ppm in its ^13^C NMR. The ^13^C NMR spectra revealed singlet signals for the carbonyl groups of 4 and 5 at approximately *δ* 166.24–167.69 ppm.

Moreover, the treatment of 3a with carbon disulphide in pyridine furnished the corresponding 2,4-dithioxo-1,2,3,4-tetrahydrothieno[2,3-*d*]pyrimidine derivative 7. IR spectrum of the latter analogue exhibited different characteristic bands at 3336–3112 cm^−1^ due to NH_2_ and 2NH and at 1682 cm^−1^ assignable to CO group. ^13^C NMR spectrum of compound 7 exhibited C=O and C=S functionalities at *δ* 164.35 and 182.43 ppm, respectively.

Compound 3a was further subjected to the reaction with thiourea and urea in refluxing ethanolic sodium ethoxide to afford the corresponding 2-thioxo/oxo-1,2-dihydrothieno[2,3-*d*]pyrimidine molecules 8a,b. IR spectra of 8a,b exhibited various bands at 3405–2210 cm^−1^ referring to 2NH_2_ and NH, at 1675 cm^−1^ due to CO of 8a and at 1689, 1664 cm^−1^ due to 2CO of 8b. ^13^C NMR spectra of compounds 8a,b showed singlet signals at *δ* 162.99 and 181.14 ppm assignable to C=O and C=S groups.

From a mechanistic perspective, the creation of thienopyrimidine nucleus of compounds 4 and 5 as sample instances is believed to follow a multi-step route. Firstly, the carbonium ion was attacked by the lone pair of NH_2_ electrons through a nucleophilic reaction, resulting in the formation of the non-isolable intermediate A. The nitrile function on the enolic OH was attacked nucleophilically to produce the second non-isolable intermediate C. After that, the intermediate C underwent intramolecular rearrangement, which yielded the new thienopyrimidine derivatives 4 and 5 (Scheme 3).

The third scheme (Scheme 4) involved treating 3a ethyl cyanoacetate and/or malononitrile in ethyl alcohol with a few amount of triethylamine to form the corresponding thieno[2,3-*b*]pyridine-5-carbonitriles 9 and 10. Furthermore, treating 3a with ethyl acetoacetate in the same way led to the production of the corresponding 5-acetyl-thieno[2,3-*b*]pyridine derivative 11. The IR spectrum of 9 exhibited a broad band at 3399 cm^−1^ corresponding to the OH group, besides the characteristic bands at 3246 and 3137 cm^−1^ due to NH_2_ groups and at 2221–2216 cm^−1^ assignable to the CN group. Also, two bands at 1715 and 1695 cm^−1^, indicating two C=O functions, were observed in IR spectrum of 11. The singlet signal summed for the three protons of the 5-COCH_3_ group was visible in the ^1^H NMR spectrum of 11, and it was detected at *δ* 22.26 ppm in the ^13^C NMR spectrum, along with the expected carbons that appeared at their expected regions.

Target compounds' mass spectra revealed molecular ion peaks that matched their molecular formulae.

**Scheme 5:**
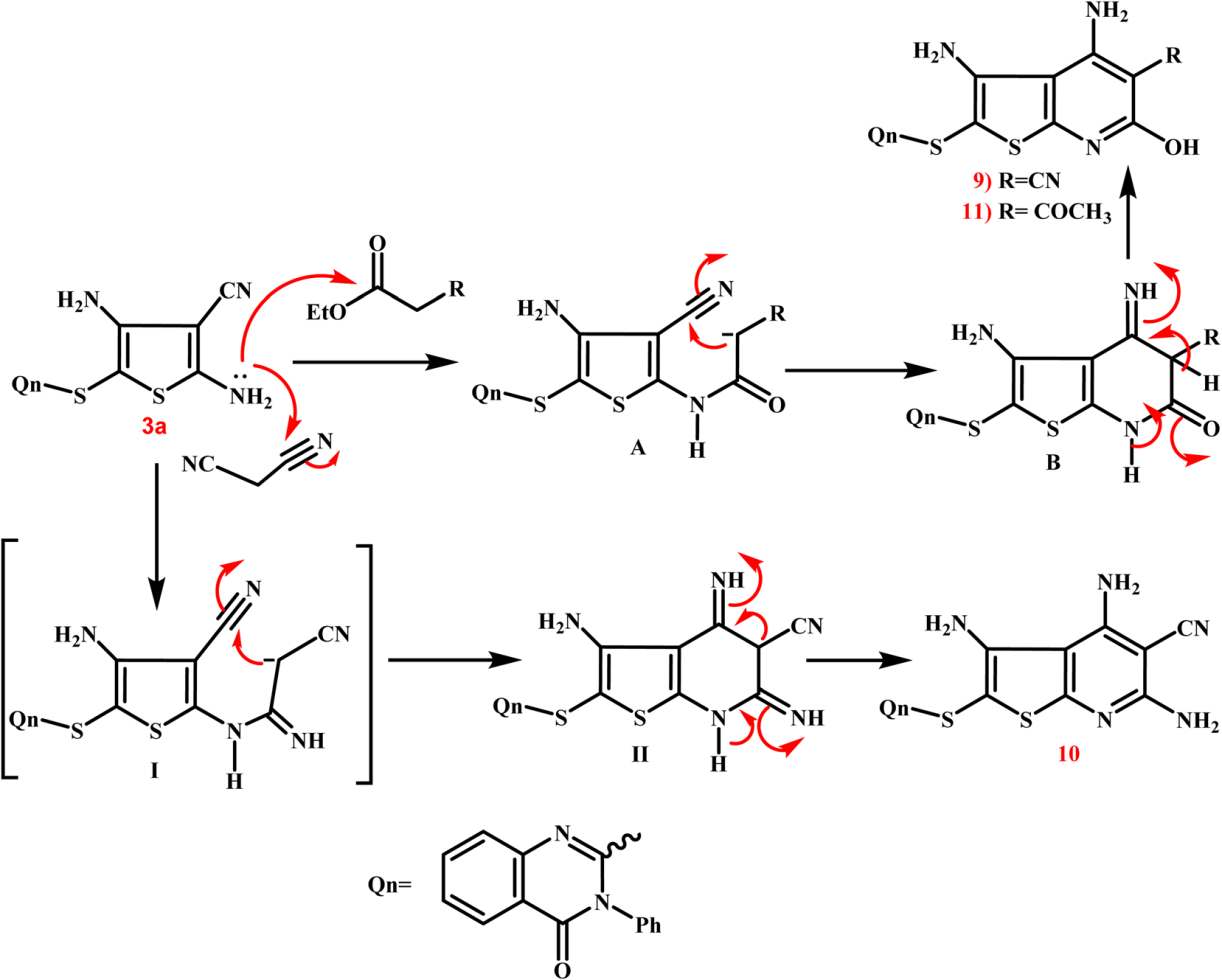
Presents the expected multi-step molecular approach for the synthesis of the novel thienopyridine derivatives 9–11.

Scheme 5 presents the expected multi-step molecular approach for the synthesis of the novel thienopyridine derivatives 9–11.

### 3.2. Biological evaluation

#### 3.2.1. *In vitro* cytotoxic activity

**Fig. 4:**
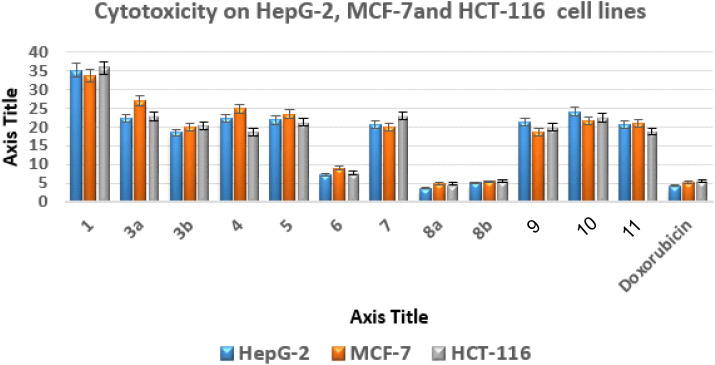
Anticancer activities of new analogues against various cell lines expressed as IC_50_ values.

The newly synthesized derivatives' *in vitro* antitumor activities were evaluated using the standard MTT assay on human normal lung fibroblast cells (WI-38) and three cancer cell lines: liver carcinoma (HepG-2), breast carcinoma (MCF-7), and colorectal carcinoma (HCT) cell lines.^51,52^ The anticancer reference control was doxorubicin. Growth suppression concentration (IC_50_) values were obtained after a 24 hour incubation period. These values indicate the concentration needed to cause 50% growth suppression (Table S1† and Fig. 4).

The most promising anticancer potential was demonstrated by thieno[2,3-*d*]pyrimidines 6, 8a, and 8b, according to the data. The 4,5-diamino-2-thioxo-1,2-dihydrothieno[2,3-*d*]pyrimidine derivative 8a demonstrated superior potency against the examined lines with IC_50_ values of 3.68 ± 0.3, 4.92 ± 0.45, and 4.88 ± 0.12 μM compared to doxorubicin with IC_50_ values of 4.44 ± 0.01, 5.20 ± 0.11, and 5.63 ± 0.11 μM, respectively.

Regarding the 2-oxo analogue 8b, it was approximately equivalent to doxorubicin with IC_50_ values of 5.13 ± 0.15, 5.44 ± 0.7, and 5.65 ± 0.37 μM. On the other hand, the 4,5-diamino-1,2-dihydrothieno[2,3-*d*]pyrimidine derivative 6 exhibited IC_50_ values of 7.22 ± 0.08, 9.14 ± 0.19, and 7.75 ± 0.3 μM against the examined cancer cells, indicating a slight decrease in activity about 2-fold less than doxorubicin.

In addition, the effects of compounds 6, 8a, and 8b on human WI-38 normal cells were evaluated, and their safety profiles were determined by calculating their selectivity indices (SI)^53,54^ (Table S1†).

Compound 8a had the highest level of safety and selectivity (SI *>* 23.12–17.29) when applied to all evaluated cancer cell lines, with compound 8b coming in second, which exhibited SI *>* 16.64–15.11, whereas compound 6 displayed SI *>* 11.12–8.80. Therefore, it is possible to draw the conclusion that compound 8a exhibits the greatest incidence of cytotoxicity among all the assessed malignant cells, with a great safety profile and excellent selectivity.^55^

#### 3.2.2. p38α MAPK inhibitory assessment

Targeting p38α MAP kinase with pharmacological inhibition may be a novel strategy for treating aggressive invasive cancers,^3,14^ due to the up-regulation of this kinase in these cases. Therefore, using SB 202190 as a reference medication, the compounds that showed the most effectiveness as cytotoxic agents against the previously tested cancer cell lines 6, 8a, and 8b were chosen as exemplary cases to examine their inhibitory effect on p38α MAP kinase. The acquired data reported as IC_50_ (μM) is shown in Table 1.

**Table 1:** Evaluation of the selected thieno[2,3-*d*]pyrimidine compounds' inhibitory activities 6, 8a, and 8b in comparison with SB-202190 against p38αMAPK^a^

Compound no.	IC_50_ (μM) (mean ± SEM)
	p38α MAPK
6	0.31 ± 0.04
8a	0.18 ± 0.02
8b	0.23 ± 0.05
SB 202190	0.27 ± 0.06

a IC_50_: compound concentration needed to 50% block the activity of the enzyme, represented by the standard error of mean (SEM): each value represents the average of three readings.

All three assessed analogues demonstrated potent p38α MAPK suppression. IC_50_ value of 4,5-diamino-2-thioxo derivative 8a was 0.18 ± 0.02 μM, indicating 1.5 times more powerful action than the reference medication of 0.27 ± 0.06 μM. When the 2-thioxo moiety was switched out for the 2-oxo moiety, the effect of analogue 8b was a little weaker than that of 8a, but it was still stronger than SB 202190 (IC_50_ = 0.23 ± 0.05 μM). Conversely, the unsubstituted thieno[2,3-*d*]pyrimidine ring at position 2 as 4,5-diamino-1,2-dihydrothieno[2,3-*d*]pyrimidine derivative 6 exhibited a further slight decrease of 1.14 fold in the inhibitory impact on p38αMAPK (IC_50_ = 0.31 ± 0.04 μM).

The compounds provided encouraging outcomes in enzymatic assessment as well as having the greatest antiproliferation efficacy and selectivity in the cell line studies. As a result, the thieno[2,3-*d*]pyrimidine fragment serves as a valuable scaffold for developing new congeners that target the p38α MAPK suppression effect as antiproliferative agents.

#### 3.2.3. The impacts of compounds 6, 8a, and 8b on levels of caspase-3, Bcl-2, and Bax, that are signs of induced apoptosis

The cell is regulated by two primary processes during the apoptotic process. There are two pathways: the extrinsic pathway, which is triggered by the death receptor, and the intrinsic pathway, which is regulated by the mitochondria. Bcl-2 protein family members synchronize the process of mitochondrial apoptosis. Two of these proteins that control this pre-programmed process are Bcl-2 and Bax; Bcl-2 is an anti-apoptotic and Bax is an inducer (pro-apoptotic). The ability of a cell to undergo apoptosis is determined by the exact balance between these two proteins.^56,57^

Also, programmed cell death (apoptosis) cannot begin until the Cysteinyl Aspartate-Specific Proteinase family, which includes caspases-3, is activated. Caspases-3 are crucial for every step of the apoptotic process, including DNA fragmentation, chromatin condensation, and cell shrinkage.^58,59^

Using the ELISA method, it was interesting to see how compounds 6, 8a, and 8b changed the amounts of caspase-3, Bcl-2, and Bax within MCF-7 carcinoma cells.^60,61^ These cells were treated with 6, 8a, and 8b at their IC_50_ concentrations (9.14, 4.92, and 5.44 μM, respectively) for 24 hours. The Bax level in the treated MCF-7 cells with the latter investigated drugs was measured in Pg mL^−1^, whereas the levels of Bcl-2 and caspase-3 were assessed in ng mL^−1^.^62^

The results showed that the three tested derivatives boosted the levels of Bax (by ∼7.31, 13.8 and 8.86 fold) and caspase 3 (by ∼3.55, 4.22 and 3.87 fold) with 6, 8a, and 8b, respectively, and decreased the level of the antiapoptotic protein Bcl-2 by ∼1.99, 3.69 and 2.66 fold, respectively in treated MCF-7 cells compared with the untreated ones. This outcome demonstrated that the compounds 6, 8a, and 8b had accelerated the cells' transition to apoptosis (Table 2).

**Table 2:** Compounds 6, 8a and 8b's influence on caspase-3, Bcl-2, and Bax levels

Compd no.	Casp-3	Bax	Bcl-2
	Conc. ng mL^−1^	Conc. Pg mL^−1^	Conc. ng mL^−1^
6/MCF-7	17.85 ± 0.22	172.31 ± 2.60	3.42 ± 0.20
8a/MCF-7	33.68 ± 0.42	205.30 ± 2.91	1.84 ± 0.15
8b/MCF-7	21.63 ± 0.15	188.25 ± 1.47	2.55 ± 0.31
Cont./MCF-7	2.44 ± 0.05	48.60 ± 1.39	6.79 ± 0.20

#### 3.2.4. Cell cycle distribution assay

Cell cycle arrest is a process that, when DNA damage occurs, stops DNA replication and disables DNA repair. This triggers the apoptotic cascade, which eventually results in the death of cancer cells.^63^ Thus, using the (PI) propidium iodide flow cytometry test, compound 8a—the most potent p38α MAPK kinase inhibitor and one that has an adequate activity as a cytotoxic agent—was selected to investigate its impact on MCF-7 cell cycle stages in contrast to untreated MCF-7 cancer cells.^64^

**Fig. 5:**
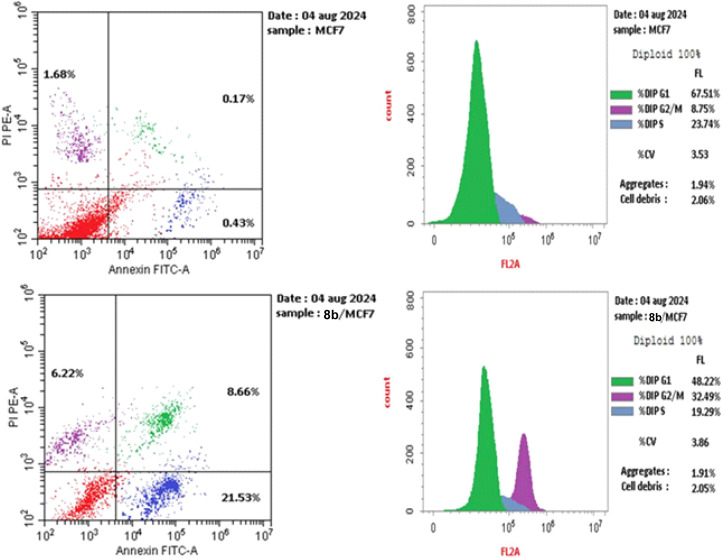
Using the annexin V/PI method, an analysis of the cell cycle and apoptosis induction of untreated control MCF-7 cell line (A) and 8a (B).

**Fig. 6:**
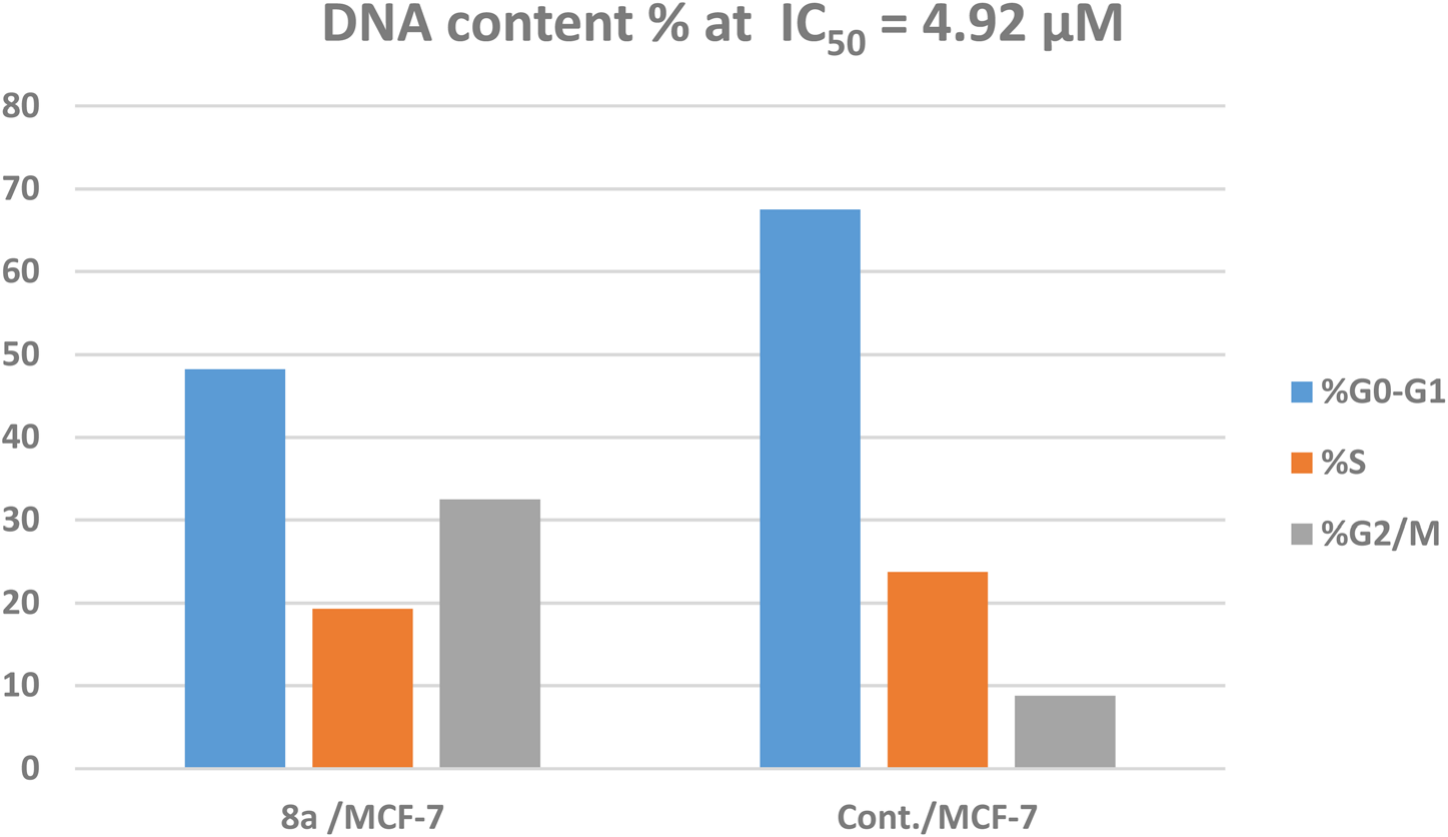
Cell cycle evaluation of the MCF-7 cell line treated with 8a.

The treatment of MCF-7 cells with 8a with its IC_50_ value of 4.68 μM for 24 h changes the normal cell cycle distribution of MCF-7 cells by increasing the amount of DNA in the G2/M phase by about four times with value of 32.49% and decreasing the amount of DNA in the G0–G1 48.22% and S phases 19.29% compared to MCF-7 cells that had not been treated with values 8.78%, 67.51%, 23.74%, respectively (Fig. 5 and 6). The above results suggest that compound 8a's antitumor mechanism of action could involve inducing apoptosis as a consequence of cell cycle arrest.

#### 3.2.5. Cell apoptosis

Using 8a at its IC_50_ value of 4.68 μM, MCF-7 cells were treated for 24 hours in order to investigate its apoptotic effect. Translocated phosphatidylserine (PS), an indicator of apoptosis, was detected with the aid of the annexin-V/Propidium Iodide (PI) double staining flow cytometry assay. FITC, annexin V, and PI were used to label the cells and those in a late stage of apoptosis were indicated by positive staining for annexin V/PI, which means that their membranes were no longer intact.^64^Fig. 6 displays the dot pattern flow cytometry data for the labeled cells.

The biparametric cytofluorimetric analysis histogram shows the division of cells into four categories: necrotic (upper left side), late apoptosis (upper right side), early apoptosis (lower right side), and viable (lower left side).

It could be noticed that compound 8a enhanced the late apoptotic cell death from 0.17% for the control to 8.66% and induced necrosis of about 6.22%. Compound 8a induced a higher late apoptosis percentage than the early phase, making the recovery of apoptotic cells to safe ones more challenging. This result confirms that apoptosis is one of 8a antiproliferative activity's main modes of action (Fig. 5 and 6).

### 3.3. Computational studies

#### 3.3.1. Docking application

Given the promising inhibition of p38α MAPK kinase by 4-oxo-3-phenylquinazoline-based candidates 6, 8a, and 8b and to estimate the probable binding affinities of these candidates, a docking simulation was performed using version 2014.0901 MOE (Molecular Operating Environment) software.^65–67^ Protein data bank was accessed to obtain the X-ray crystallographic structure of p38α MAPK and its native ligand, 4-[3-methylsulfanylanilino]-6,7-dimethoxyquinazoline MSQ (PDB code: 1DI9).^68^ Initially, we re-docked the native ligand MSQ within its receptor to confirm the docking process. This allowed for an energy score of −11.36 kcal mol^−1^ and a tiny RMSD value of 0.79 Å between the docked pose and the co-crystallized ligand. According to ^68^ and shown in Fig. S1,† the backbone of Met109 in p38α MAPK formed a hydrogen bond with nitrogen at position 1 of MSQ. Furthermore, Thr106 was coupled to the nitrogen at position 3 through a hydrogen bond with a water molecule that was buried there.

**Fig. 7:**
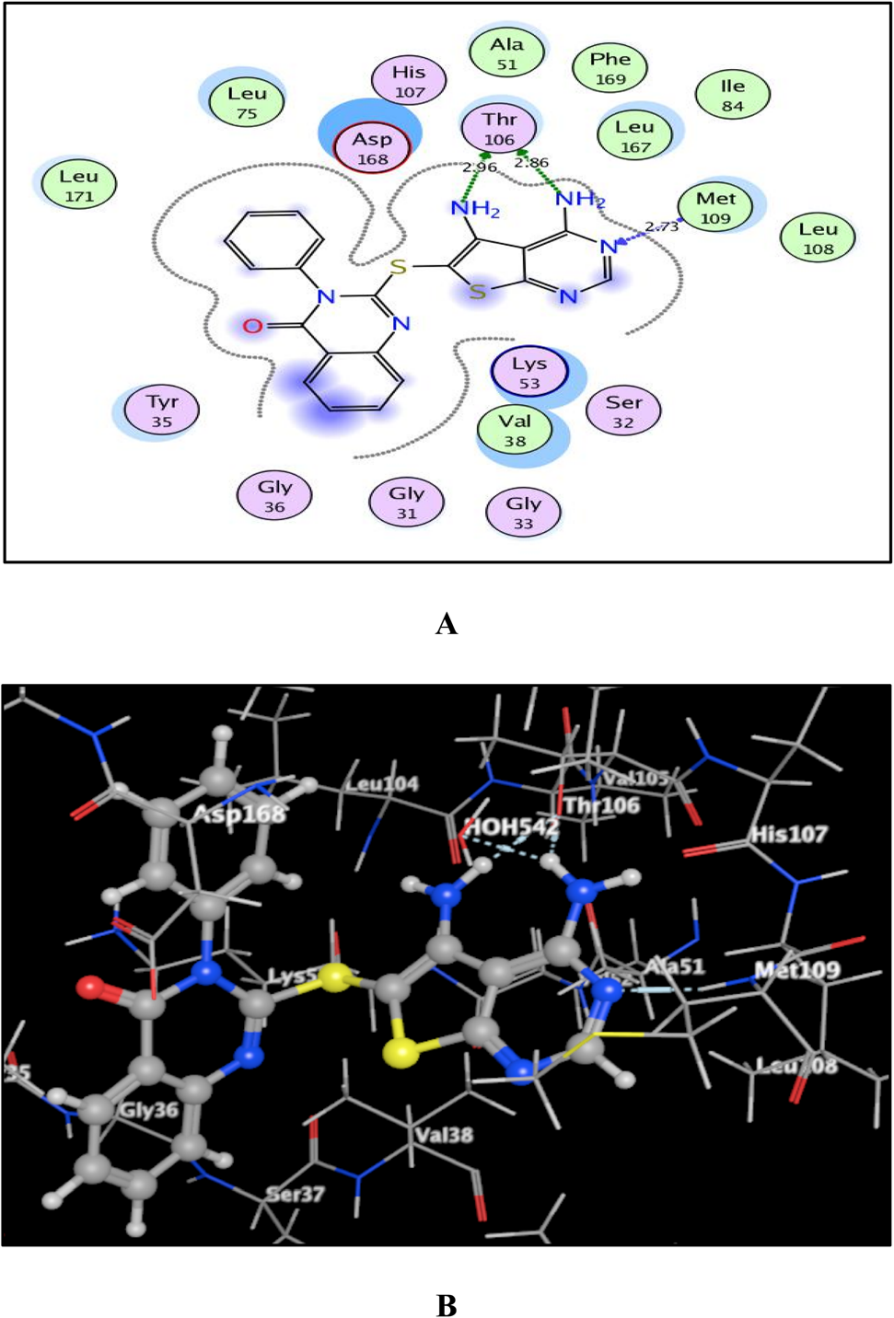
(A) and (B) patterns demonstrate two and three dimensional binding poses of the promising 4,5-diaminothieno[2,3-*d*]pyrimidine, 6 in the active pocket of p38α MAPK (PDB ID: 1DI9).

**Fig. 8:**
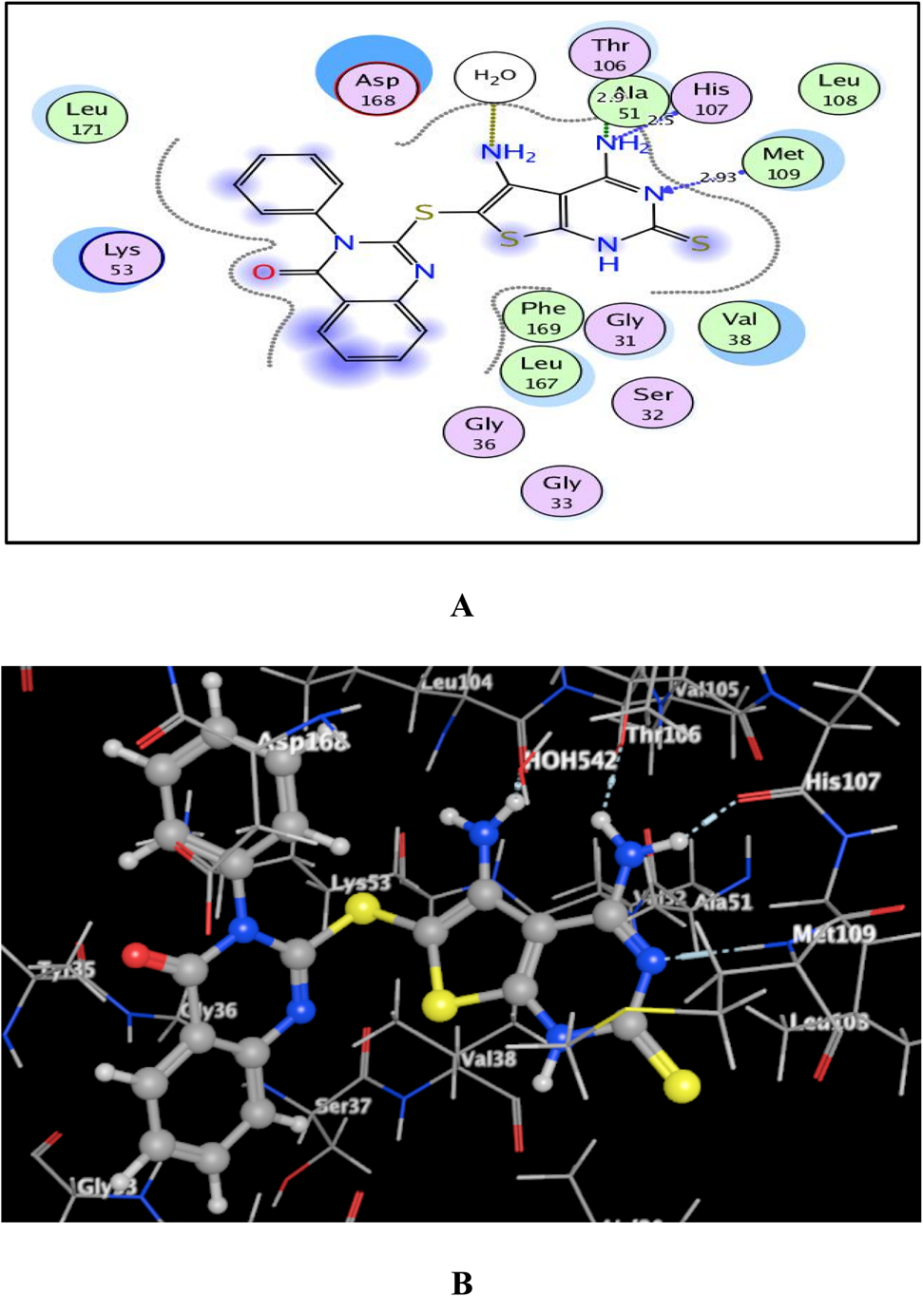
(A) and (B) views demonstrate two and three dimensional binding poses of promising 8a in the active pocket of p38α MAPK (PDB ID: 1DI9).

**Fig. 9:**
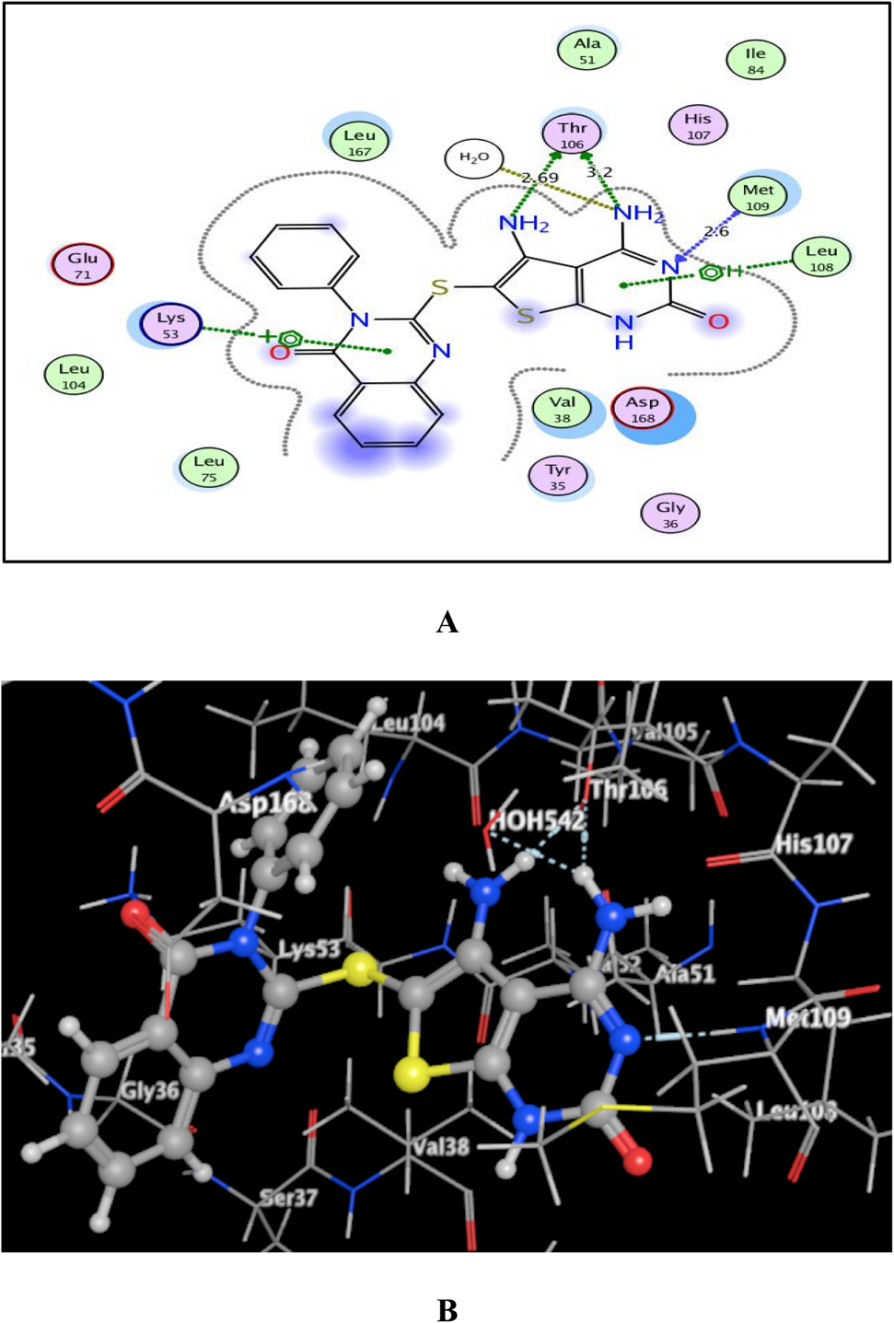
(A) and (B) views demonstrate two and three dimensional binding poses of promising 8b in the active pocket of p38α MAPK (PDB ID: 1DI9).

As shown in Fig. 7–9, all 4-oxo-3-phenylquinazoline-based candidates 6, 8a, and 8b were embedded nicely within p38α MAPK with significant energy scores of −10.88, −11.28, and −10.96 kcal mol^−1^, respectively. There were H-bond acceptors between N3 and the backbone of Met109 in all of the screened thieno[2,3-*d*]pyrimidines 6, 8a, and 8b. Also, in derivatives 6 and 8b, Thr106's side chain and the two amino groups at p-4 and p-5 formed two hydrogen bond donors. However, in 8a, the amino group at position 4 generated two hydrogen bond donors with Thr106 side chain and His107 backbone the, which were separated by 2.90 and 2.5 Å, respectively (Fig. 8 and 9). Furthermore, the amino groups at p-4 in 8b and p-5 in 8a formed ionic bonds with the water molecule HOH-42. The target 8b revealed further hydrophobic interactions between quinazoline and thieno[2,3-*d*]pyrimidine moieties with Lys53 and Leu108, respectively.

Based upon earlier observations, the presence of a quinazoline scaffold with a thieno[2,3-*d*]pyrimidine core strengthened and sustained the fixation of 6, 8a and 8b within the binding pocket of p38α MAPK. The incorporation of NH_2_ at p-4 of thieno[2,3-*d*]pyrimidine was found to enhance binding with the important amino acid Thr106, resulting in enhanced inhibitory activity. Accordingly, the thieno[2,3-*d*]pyrimidines 6, 8a, and 8b should be looked at as possible leads for more research and design to make anticancer medications that work very well at stopping the growth of cancer cells.

#### 3.3.2. *In silico* ADMET prediction

The attractive 4-oxo-3-phenylquinazoline-based candidates 6, 8a, and 8b were further examined for their anticipated pharmacokinetic, physicochemical properties, and toxicity using the free websites SwissADME and admetSAR 1.0.^69–72^

Analyzing ADMET (absorption, distribution, metabolism, excretion, and toxicity) of the targeted drugs might yield crucial information on the best medication choice.

The utilization of free internet resources SwissADME and AdmetSAR 1.0 allowed for the completion of this eagerly anticipated study.^69–72^ Veber and Lipinski's guidelines could be used to determine which medication behaves best when taken orally. With one exception to Veber rule (TPSA > 140), it was demonstrated that thieno[2,3-*d*]pyrimidine-based candidates 6, 8a and 8b under examination complied with the prior regulations (Table 3).

**Table 3:** Calculated physicochemical characteristics of attractive thieno[2,3-*d*]pyrimidines 6, 8a and 8b

Compd	MW^a^	TPSA^b^ (Å^2^)	nRB^c^	nHBA^d^	nHBD^e^	*M* log *P*^f^	Violations^g^
Rule	≤500	≤140	≤10	≤10	≤5	≤4.15	—
6	418.49	166.25	3	4	2	2.58	0 (Lipinski)
							1 (Veber)
							TPSA > 140
8a	450.56	201.24	3	3	3	2.51	0 (Lipinski)
							1 (Veber)
							TPSA > 140
8b	434.49	186.22	3	4	3	2.53	0 (Lipinski)
							1 (Veber)
							TPSA > 140

a Molecular weight.

b Topological polar surface area.

c Number of rotatable bond.

d Number of hydrogen bond acceptor.

e Number of hydrogen bond donor.

f Calculated lipophilicity (*M* log *P*_o/w_).

g Violations from Lipinski and Veber rules.

**Fig. 10:**
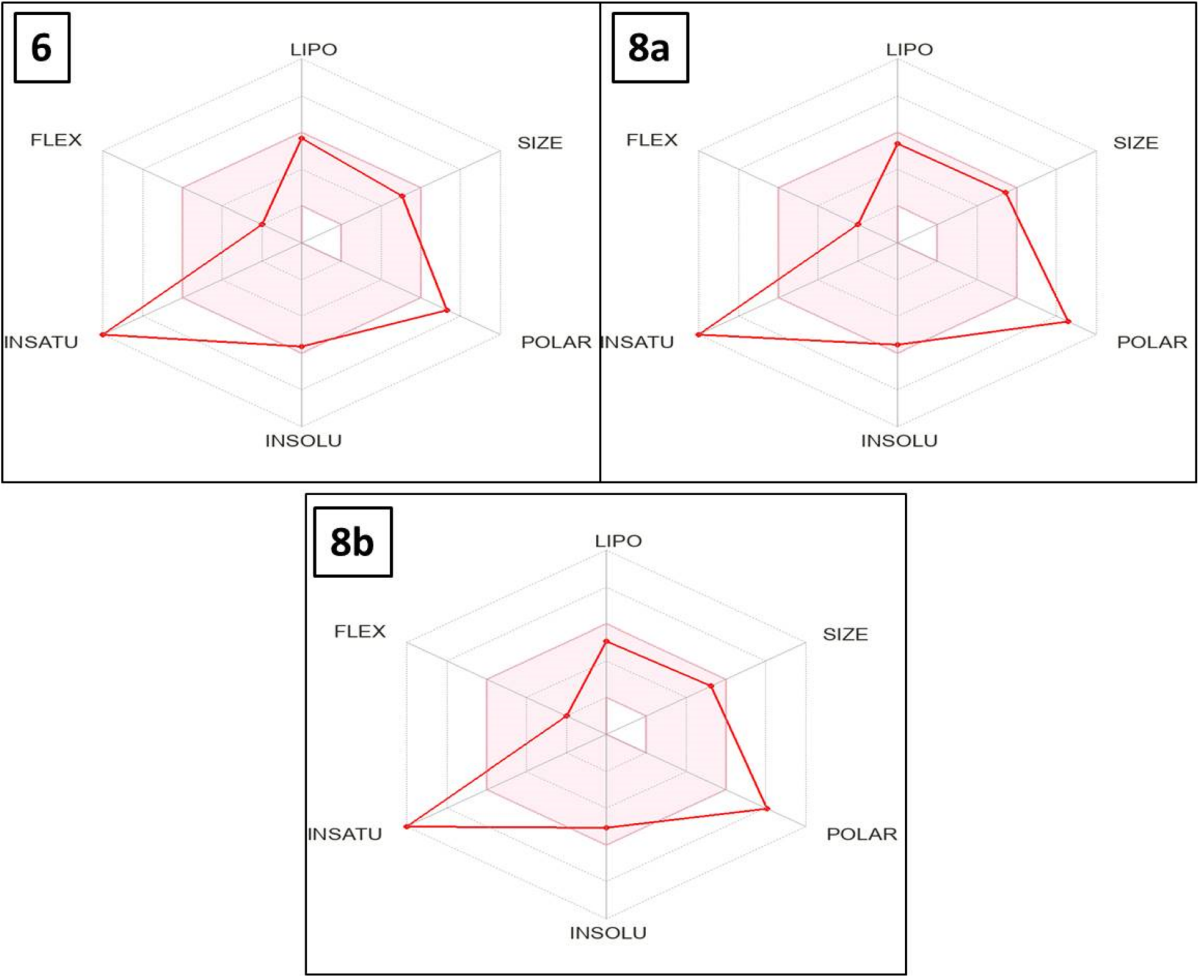
The effective thieno[2,3-*d*]pyrimidine-based derivatives 6, 8a and 8b's bioavailability radar map. The optimal values for each component of oral bioavailability are represented by the pink region, whereas the red lines indicate the expected values for the studied targets.

The bioavailability radar map indicates that, with the exception of polarity and saturation, the evaluated thieno[2,3-*d*]pyrimidines 6, 8a and 8b were in the perfect range (pink region) of the critical variables (solubility, lipophilicity, flexibility and size) (Fig. 10). Thus, these studies provided substantial proof concerning the oral bioavailability of the investigated compounds.

Table S6† presents an admetSAR 1.0 study of ADMET linked to putative thieno[2,3-*d*]pyrimidine-based targets 6, 8a and 8b. With these compounds, there was a greater probability of gastrointestinal absorption without any blood–brain barrier penetration. Therefore, they might be utilized to remedy disorders at the periphery without causing central complications. Drug efflux transporter P-gp (P-glycoprotein) is responsible for removing medicines from cells and may have an impact on drug tolerance. The thieno[2,3-*d*]pyrimidines 6, 8a and 8b, which were screened, exhibit little discharge from cells with highest activity since they are not P-gp substrates.

Quinazoline-thieno[2,3-*d*]pyrimidine-based derivatives 6, 8a and 8b are distributed and concentrated within the mitochondria. Indeed, studies have indicated that inhibiting more CYP enzymes increases the possibility that a medication would interact with other active molecules in drug–drug interactions (DDI).^73^ As a result, it was anticipated that these drugs would have little effect on the majority of CYPs.

All screened targets did not inhibit the potassium channel related to the human ether-a-go-go gene (hERG) as predicted. This implies that there might not be much of a chance for cardiac side effects or cardiotoxicity, which are frequent worries during pharmaceutical candidate clinical trials. Thieno[2,3-*d*]pyrimidines 8a and 8b exhibited no Ames toxicity, implying no risk of genotoxicity, whereas 6 did not. Moreover, thieno[2,3-*d*]pyrimidines 6, 8a and 8b provided readings ranging from 617.6 to 688.7 mg kg^−1^ based on estimations of acute oral toxicity. Since these results fell into the third category (500 mg kg^−1^ < LD_50_ ≤ 5000 mg kg^−1^), they were categorized as harmless substances. The carcinogenicity descriptor (CARC) values of these chemicals, which varied from 424.3 to 448.5 mg per kg body weight per day, indicate that they may also be categorized as non-required and non-carcinogenic. It was expected that these derivatives would be deemed to be non-biodegradable materials when their capacity to break down in the environment was assessed.

## 4. Conclusion

In brief, three new series of thiophene, thieno[2,3-*d*]pyrimidine, and thieno[2,3-*b*]pyridine conjugated with 4-oxo-3-phenyl-2-thio-3,4-dihydroquinazoline scaffolds were created to evaluate their anticancer impact as p38α MAPK inhibitors. Compounds 6, 8a, and 8b exhibited the greatest anticancer efficacy against liver HepG-2, breast MCF-7, and colorectal HCT-116 carcinoma cells, which is approximately equivalent to that of doxorubicin. The latter compounds also showed encouraging safety profiles when tested against normal WI-38 cells with SI values *>*17.29, *>*15.11, and >8.80, respectively. Furthermore, compounds 6, 8a, and 8b were subjected to a p38α MAPK inhibition assay, with SB 202190 acting as the reference drug. Compounds 8a and its 2-oxo analogue 8b were more potent inhibitors than the reference drugs (IC_50_s = 0.18 ± 0.02, 0.23 ± 0.05 μM and 0.27 ± 0.06 μM, respectively). Derivative 6, on the other hand, had an IC_50_ value of 0.31 ± 0.04 μM, which was 1.14 times lower than SB 202190. Furthermore, the three promising analogues 6, 8a, and 8b boosted the levels of Bax (by ∼7.31, 13.8 and 8.86 fold, respectively) and caspase 3 (by ∼3.55, 4.22 and 3.87 fold, respectively), and decreased the level of the antiapoptotic protein Bcl-2 by ∼1.99, 2.66 and 3.69 fold, respectively in treated MCF-7 cells compared with the untreated ones. Additionally, 8a arrested MCF-7 cell cycle at G2/M phase and induced necrotic and apoptotic effects in the late stages.

Molecular docking study exhibited that the candidates 6, 8a, and 8b were embedded nicely within p38α MAPK with significant energy scores of −10.88, −11.28, and −10.96 kcal mol^−1^, respectively. Ultimately, the physico-chemical and ADMET properties of 6, 8a, and 8b were computed *in silico*. According to the calculated results, these compounds have the potential to be excellent candidates for additional development and optimization in later research.

## Data availability

The data supporting this article have been included as part of the ESI.†

## Conflicts of interest

The authors declare that they have no known competing financial interests or personal relationships that could have appeared to influence the study in this manuscript.

## Supplementary Material

RA-015-D4RA06744D-s001
