# Correction: Design, synthesis, and *in silico* studies of new quinazolinones tagged thiophene, thienopyrimidine, and thienopyridine scaffolds as antiproliferative agents with potential p38α MAPK kinase inhibitory effects

**DOI:** 10.1039/d5ra90011e

**Published:** 2025-01-27

**Authors:** Aisha A. Alsfouk, Ismail M. M. Othman, Manal M. Anwar, Asmaa Saleh, Eman S. Nossier

**Affiliations:** a Department of Pharmaceutical Sciences, College of Pharmacy, Princess Nourah bint Abdulrahman University P. O. Box 84428 Riyadh 11671 Saudi Arabia; b Department of Chemistry, Faculty of Science, Al-Azhar University Assiut 71524 Egypt; c Department of Therapeutic Chemistry, Pharmaceutical and Drug Industries Research Institute, National Research Centre El-Bohouth Street, Dokki, P. O. Box 12622 Cairo Egypt manal.hasan52@live.com; d Pharmaceutical Medicinal Chemistry and Drug Design Department, Faculty of Pharmacy (Girls), Al-Azhar University Cairo 11754 Egypt dr.emannossier@gmail.com dremannossier@azhar.edu.eg; e The National Committee of Drugs, Academy of Scientific Research and Technology Cairo 11516 Egypt

## Abstract

Correction for ‘Design, synthesis, and *in silico* studies of new quinazolinones tagged thiophene, thienopyrimidine, and thienopyridine scaffolds as antiproliferative agents with potential p38α MAPK kinase inhibitory effects’ by Aisha A. Alsfouk *et al.*, *RSC Adv.*, 2025, **15**, 1407–1424, https://doi.org/10.1039/D4RA06744D.

The authors regret that an incorrect grant number was shown in the acknowledgements section of the published article. The corrected section should read:

This research was funded by Princess Nourah bint Abdulrahman University researchers supporting project number (PNURSP2025R116), Princess Nourah bint Abdulrahman University, Riyadh, Saudi Arabia.

The Royal Society of Chemistry apologises for these errors and any consequent inconvenience to authors and readers.

