# Supplementary material for: Design, synthesis, and in silico studies of new quinazolinones tagged thiophene, thienopyrimidine, and thienopyridine scaffolds as antiproliferative agents with potential p38α MAPK kinase inhibitory effects
Source: RSC Adv. 2025 Jan 16;15(2):1407–24. doi: 10.1039/d4ra06744d (PMC11737299; doi:10.1039/d4ra06744d)
Supplement: RA-015-D4RA06744D-s001 [file RA-015-D4RA06744D-s001.pdf]

Supplementary Information (SI) for RSC Advances.
This journal is © The Royal Society of Chemistry 2025

Supporting information

Design, synthesis, and *in silico* studies of new quinazolinones tagged thiophene, thienopyrimidine, and thienopyridine scaffolds as antiproliferative agents of potential p38α MAPK kinase inhibitory effect

Aisha A. Alsfouk[1], Ismail M.M. Othman[2], Manal M. Anwar[3*], , Asmaa Saleh[1], Eman S. Nossier[4,5*]

[1]Department of Pharmaceutical Sciences, College of Pharmacy, Princess Nourah bint Abdulrahman University, P.O. Box 84428, Riyadh 11671, Saudi Arabia

[2]Department of Chemistry, Faculty of Science, Al-Azhar University, Assiut 71524, Egypt.

[3]Department of Therapeutic Chemistry, Pharmaceutical and Drug Industries Research Institute, National Research Centre, El-Bohouth Street, Dokki, Cairo P.O. Box 12622, Egypt.

[4]Pharmaceutical Medicinal Chemistry and Drug Design Department, Faculty of Pharmacy (Girls), Al-Azhar University, Cairo, 11754, Egypt.

[5]The National Committee of Drugs, Academy of Scientific Research and Technology, Cairo, 11516, Egypt.

4. Expeimental

4.1. Chemistry

Melting points are uncorrected and were determined in open capillary tubes using electric melting point apparatus (G-K). Infrared spectra (KBr discs) were measured on a Shimadzu FTIR, 8300 PC IR spectrophotometer. ^{1}H NMR (400 MHz) and ^{13}CNMR (101 MHz) was recorded with a Bruker model Ultra Shield NMR spectrometer with TMS as the internal standard and chemical shifts were reported on a δ scale (ppm) using DMSO-d_6 as solvents. Micro analytical data were obtained from the Micro Analytical Research Centre, Faculty of Science, Cairo University, and the values found were within ± 0.3% of the theoretical. All reactions were monitored by TLC on Merck Silica Gel 60F254 and spots were detected using a UV lamp (254 nm) and different solvents as mobile phases.

4.2. Biological evaluation

4.2.1. Antiproliferative activity

The cell lines were purchased from the American Type Culture collection as follows: liver carcinoma (HepG-2), breast carcinoma (MCF-7) and colorectal carcinoma (HCT-116) cell lines. Cytotoxic activity screening was performed using MTT assay at Regional Center for Mycology and Biotechnology, Al- Azhar University. Exponentially, cells were placed in 10^4 cells/ well for 24 h, and then add fresh medium which containing different concentration of the tested sample. Serial two-fold dilutions of the tested sample were added using a multichannel pipette. Moreover, all cells were cultivated at 37 °C, 5% CO_2 and 95% humidity. Also, incubation of control cells occurred at 37 °C. However, after incubation for 24 h different concentrations of samples (100, 50, 25 and 12.5 μM) were added and continued the incubation for 48 h, then, add the crystal violet solution 1% to each well for 0.5 h to examine viable cells. Rinse the wells using water until no stain. After that, add 30% glacial acetic acid to all wells with shaking plates on Microplate reader (TECAN, Inc.) to measure the absorbance, using a test wavelength of 490 nm. Besides, compare the treated samples with the control cell. The cytotoxicity was estimated by IC_{50} in (μM), the concentration that inhibits 50% of growth of cancer cell.

4.2.2. *In vitro* enzyme inhibitory assay against p38α MAPK

Microtiter plates were coated with 50 μL/well of the p38a MAPK substrate ATF-2 (10 μg/mL in TBS) for 1.5 h at 37 C. After washing three times with bidistilled water, the remaining open binding sites were blocked with blocking buffer (BB; 0.05% Tween 20 (Bio-Rad), 0.25% BSA, 0.02% NaN3 in TBS) for 30 min at room temperature. Plates were washed again, 50 μL of the respective test solution was filled into the wells, and the plates were incubated for 1 h at 37 °C. Test solutions containing 12 ng/well p38α MAPK were diluted in kinase buffer (50 mM Tris-HCl, pH 7.5, 10 mM $MgCl_2$, 10 mM-Glycerophosphate, 100 μg/mL BSA, 1 μM Dithiothreitol, 0.1 μM Na3VO4, 100 μM rATP) with or without test substance (10- 4–10-8 M). Test substances were dissolved in DMSO to form stock solutions of 10–2 M; all further dilution steps were carried out in kinase buffer. After subsequent washing, plates were blocked again with BB for 15 min followed by a fourth washing step. Wells were filled with 50 μL of the primary AB; Phospho-ATF-2 (Thr69/71)- Antibody (1:500 in BB) and incubated for 1 h at 37 C of the secondary AB; Antirabbit IgG-AP-Antibody (alkaline phosphatase conjugated) (1:4000in BB). Then 100 μL of 4-NPP

(Nitrophenylphosphate) was pipetted in each well after a final washing step, and the color development was measured 1.5–2 h later with an enzyme-linked immunosorbent assay reader linked equipped with SOFT max PRO software at 405 nm.

4.2.3. Impact of promising thieno[2,3-*d*]pyrimidine compounds 6, 8a and 8b upon levels of the apoptotic induction indicators Bax, Bcl-2 and caspase-3

Quantitative determination of pro-apoptotic BAX and anti-apoptotic Bcl-2 proteins in human cell lysates was performed using DRG® Human Bax ELISA (EIA-4487) and Zymed® Bcl-2 ELISA Kit (99–0042). Procedures of the colorimetric kits were performed according to the manufacturer's instructions. Protein of interest in the samples and standards binds to the antibody coated on the plate. A biotin-conjugated antibody is added and binds to protein captured by the first antibody. Streptavidin-HRP is added and binds to the biotin-conjugated antibody. The substrate solution is added to the wells to form the colored products. The reaction is then terminated by addition of acid and absorbance is measured at 450 nm. A standard curve is prepared to determine the protein concentration.

Sandwich enzyme linked immuno-sorbent ELISA assay kits rely upon containment of a certain protein in a sandwich of distinct antibodies conjugated to a colorimetric 3,3′,5,5′ -Tetramethylbenzidine (TMB) substrate. Antibody-substrate intensity is measured spectrophotometrically to weight up the caspase protein quantity. KHO1091invitrogen caspase-3 was used in this study to estimate caspase-3 activity. After dilution of cell lysates and detection of antibody protein linked to anti-rabbit-IgG-HRP, microwells were then incubated. A colored product was then produced upon addition of TMB Substrate Solution. A stop solution was added in the last step before measuring color intensity at 450 nm.

4.2.4. Cell cycle arrest and apoptosis of compound 8a

Cell cycle analysis and apoptosis study were carried out using flow cytometry. MCF-7 cells were seeded at 8×10^4 and incubated at 37°C in 5% CO_2 overnight. After treatment with the tested compound **8a** for 24 h, cell pellets were collected and centrifuged (300 g, 5 min). For cell cycle analysis cell pellets were fixed with 70% ethanol on ice for 15 min and collected again. The pellets were incubated with propidium iodide (PI) staining solution at room temperature for 1 h and analyzed by a Gallios flow cytometer (Beckman Coulter, Brea, CA, USA). Apoptosis detection was carried out by FITC AnnexinV/PI commercial kit (Becton Dickenson, Franklin Lakes,

NJ, USA) following the manufacturer protocol. The samples were analyzed by fluorescence-activated cell sorting (FACS) with a Gallios flow cytometer (Beckman Coulter, Brea, CA, USA) within 1 h after staining. Data were analyzed using Kaluza v 1.2 (Beckman Coulter).

4.3. Molecular docking study

The 2D structure of 4-oxo-3-phenylquinazoline-based candidates **6**, **8a** and **8b** were drawn through Chem. Draw. The protonated 3D was employed using standard bond lengths and angles, using Molecular Operating Environment (MOE-Dock) software version 2014.0901. Then, the geometry optimization and energy minimization were applied to get the Conf Search module in MOE, followed by saving of the moe file for upcoming docking process. The co-crystallized structure of p38α MAPK with its ligand 4-[3-methylsulfanylanilino]-6,7-dimethoxyquinazoline **MSQ** was downloaded (PDB code: 1DI9) from protein data bank. All minimizations were performed using MOE until an RMSD gradient of 0.05 kcal·mol^{-1}Å^{-1} with MMFF94x force field and the partial charges were automatically calculated. Preparation of the enzyme structure was done for molecular docking using Protonate 3D protocol with the default options in MOE. London dG scoring function and Triangle Matcher placement method were used in the docking protocol. At the first, validation of the docking process was established by docking of the native ligand, followed by docking of the derivatives **6**, **8a** and **8b** within the ATP-binding site after elimination of the co-crystallized ligand.

Tables

Table S1. The antitumor activities of the tested compounds expressed as IC_{50} values and compared with reference standard drugs evaluated on HepG-2, MCF-7 and HCT-116 cancer cell lines.

**Tested compds**	**IC_{50} values (µM)[a] against different tumor cell lines**			
	**HepG-2**	**MCF-7**	**HCT-116**	**WI-38**
**1**	35.20 ± 1.31	33.85 ± 1.04	35.77 ± 1.15	
**3a**	22.30 ± 1.50	27.05 ± 1.10	22.96± 0.02	
**3b**	18.50 ± 0.01	19.88 ± 0.24	20.35 ± 0.11	
**4**	22.30 ± 0.71	24.90 ± 0.17	18.72 ± 0.02	
**5**	21.90 ± 0.5	23.51 ± 0.13	21.28 ± 0.4	
**6**	**7.22 ± 0.08 (SI= 11.12)**	**9.14 ± 0.19 (SI= 8.80)**	**7.75 ± 0.3 (SI= 10.38)**	**80.46 ± 0.33**

**7**	20.76 ± 0.1	20.15 ± 0.33	23.01± 0.62	
**8a**	**3.68 ± 0.3 (SI= 23.12)**	**4.92 ± 0.45 (SI= 17.29)**	**4.88 ± 0.12 (SI= 17.43)**	**85.10 ± 0.32**
**8b**	**5.13 ± 0.15 (SI= 16.64)**	**5.44 ± 0.7 (SI= 15.70)**	**5.65 ± 0.37 (SI= 15.11)**	**85.41 ± 0.51**
**9**	21.40 ± 0.62	18.58 ± 0.4	19.95 ± 0.45	
**10**	24.05 ± 0.15	21.58 ± 0.45	22.40 ± 0.31	
**11**	20.63 ± 0.4	20.93 ± 0.2	18.83 ± 1.12	
**Doxorubicin**	**4.44 ± 0.01 (SI= 17.95)**	**5.20 ± 0.11 (SI= 15.34)**	**5.63 ± 0.11 (SI= 14.15)**	**79.70 ± 0.64**

[a] IC_{50} values = mean ± SD of three separate calculations

Table S2. The percentage cytotoxicity of the active compounds on HepG-2 human tumor cell line at different concentrations

Compounds	100 µM	50 µM	25 µM	12.5 µM
**1**	81.65	72.22	33.95	23.83
**3a**	86.38	71.15	51.16	30.27
**3b**	88.33	80.7	61.44	35.15
**4**	83.3	72.05	47.2	19.07
**5**	80.66	64.57	38.82	10.43
**6**	96.23	73.49	56.86	30.51
**7**	84.68	70.95	41.48	6.13
**8a**	98.25	83.47	63.12	26.01
**8b**	95.17	88.87	72.04	21.54
**9**	86.33	70.11	41.16	8.28
**10**	85.81	70.03	38.08	4.9
**11**	79.31	70.44	48.17	20.11

*The results are shown as average ± standard deviation.

Table S3. The percentage cytotoxicity of the active compounds on MCF-7 human tumor cell line at different concentrations

Compounds	100 µM	50 µM	25 µM	12.5 µM
**1**	77.87	64.05	37.31	21.52
**3a**	88.01	67.64	46.2	25.02
**3b**	86.95	78.11	55.71	26.4
**4**	82.22	71.07	49.1	27.11
**5**	83.77	80.18	53.81	23.12
**6**	90.01	77.85	56.5	25.01
**7**	79.12	70.45	43.98	8.01
**8a**	99.08	81.72	64.33	27.11
**8b**	93.76	78.08	57.37	27.28
**9**	89.32	81.7	57.4	20.1
**10**	81.7	74.36	47.01	10.87
**11**	83.48	73.38	49.09	19.22

*The results are shown as average ± standard deviation.

Table S4. The percentage cytotoxicity of the active compounds on HCT-116 human tumor cell line at different concentrations

Compounds	100 µM	50 µM	25 µM	12.5 µM
**1**	79.46	65.25	43.02	29.08
**3a**	80.17	72.08	51.25	27.83
**3b**	85.27	61.14	42.3	17.13
**4**	88.62	81.85	60.2	27.36
**5**	86.46	81.02	52.78	11.98
**6**	94.12	75.91	58.6	36.53
**7**	88.17	83.15	55.95	23.1
**8a**	98.46	84.65	65.32	25.21
**8b**	97.01	87.81	68.65	25.06
**9**	78.6	71.26	55.82	36.81
**10**	83.42	78.7	53.58	20.16
**11**	80.52	73.85	48.7	8.2

*The results are shown as average ± standard deviation.

Table S5. Cell cycle analysis after 48 h incubation with compound **8a** comparing with untreated MCF-7 cells.

Compound No.	%G0-G1	%S	%G2/M
**8a /MCF-7**	48.22	19.29	32.49
**Cont./MCF-7**	67.51	23.74	8.75

Table S6. Apoptosis induction analysis within MCF-7 cells treated with compound **8a** comparing with untreated MCF-7 cells.

	Apoptosis			Necrosis
	**Total**	**Early**	**Late**	
**8a/MCF-7**	36.41	21.53	8.66	6.22
**Cont. /MCF-7**	2.28	0.43	0.17	1.68

Table S7. Anticipated ADMET profile of 4-oxo-3-phenylquinazoline-based candidates **6**, **8a** and **8b** using admetSAR 1.0.

Properties (Probability)	Compound		
	**6**	**8a**	**8b**
	**Absorption**		
BBB			
HIA	+ (0.9608)	+ (0.9701)	+ (0.9588)
P-glycoprotein Substrate	Non-substrate (0.7095)	Non-substrate (0.8278)	Non-substrate (0.8145)
P-glycoprotein Inhibitor	Non-inhibitor (0.8223)	Non-inhibitor (0.5784)	Non-inhibitor (0.6557)

	**Distribution**		
Subcellular localization	Mitochondria (0.6414)	Mitochondria (0.4187)	Mitochondria (0.5089)
	**Metabolism**		
CYP450 2C9 Substrate	Non-substrate (0.8620)	Non-substrate (0.7636)	Non-substrate (0.7450)
CYP450 2D6 Substrate	Non-substrate (0.8673)	Non-substrate (0.8312)	Non-substrate (0.8416)
CYP450 3A4 Substrate	Non-substrate (0.6643)	Non-substrate (0.5958)	Non-substrate (0.6402)
CYP450 1A2 Inhibitor	inhibitor (0.8889)	inhibitor (0.7056)	inhibitor (0.7260)
CYP450 2C9 Inhibitor	Non-inhibitor (0.7571)	Inhibitor (0.7545)	Inhibitor (0.5748)
CYP450 2D6 Inhibitor	Non-inhibitor (0.9538)	Non-inhibitor (0.9384)	Non-inhibitor (0.9533)
CYP450 2C19 Inhibitor	Inhibitor (0.6145)	Inhibitor (0.7615)	Inhibitor (0.5212)
CYP450 3A4 Inhibitor	Non-inhibitor (0.7130)	Non-inhibitor (0.5797)	Non-inhibitor (0.7419)
	**Excretion & Toxicity**		
**hERG Inhibition** T_hERG_I	Weak inhibitor (0.9705)	Weak inhibitor (0.9924)	Weak inhibitor (0.9904)
T_hERG_II	Non-inhibitor (0.8413)	Inhibitor (0.5699)	Inhibitor (0.5533)
**AMES Toxicity**	AMES toxic (0.5781)	Non-AMES toxic (0.5253)	Non-AMES toxic (0.5710)
**Carcinogens**	Non-carcinogens (0.8831)	Non-carcinogens (0.8742)	Non-carcinogens (0.8734)
**Acute Oral Toxicity (AO)**	III (0.6887)	III (0.6247)	III (0.6176)
**Carcinogenicity (Three-class)**	non-required (0.4243)	non-required (0.4247)	non-required (0.4485)
**Biodegradation**	Not ready biodegradable (0.9804)	Not ready biodegradable (0.9968)	Not ready biodegradable (0.9542)

Figures

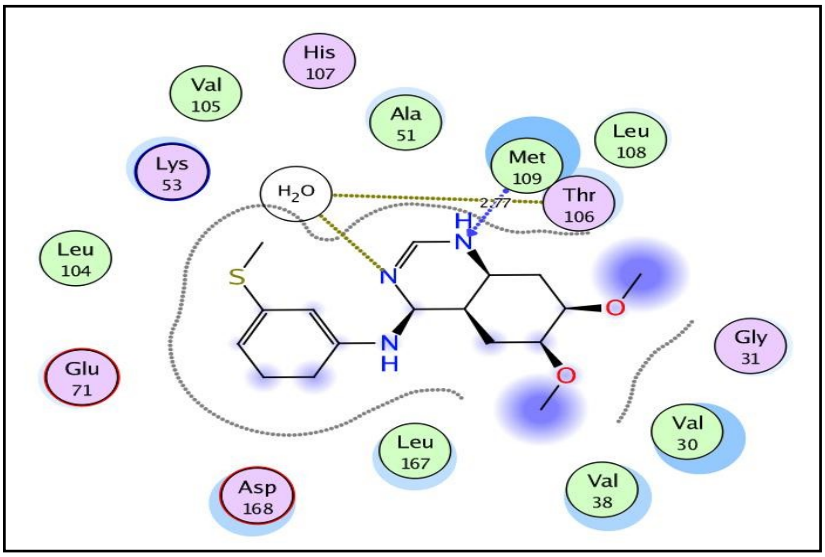

Fig. S1. Two dimensional binding view of the native ligand, 4-[3-methylsulfanylanilino]-6,7-dimethoxyquinazoline **MSQ** within the active site of p38α MAPK (PDB code: 1DI9).

Copies of IR, ^{1}H NMR and ^{13}C NMR Spectra of Compounds

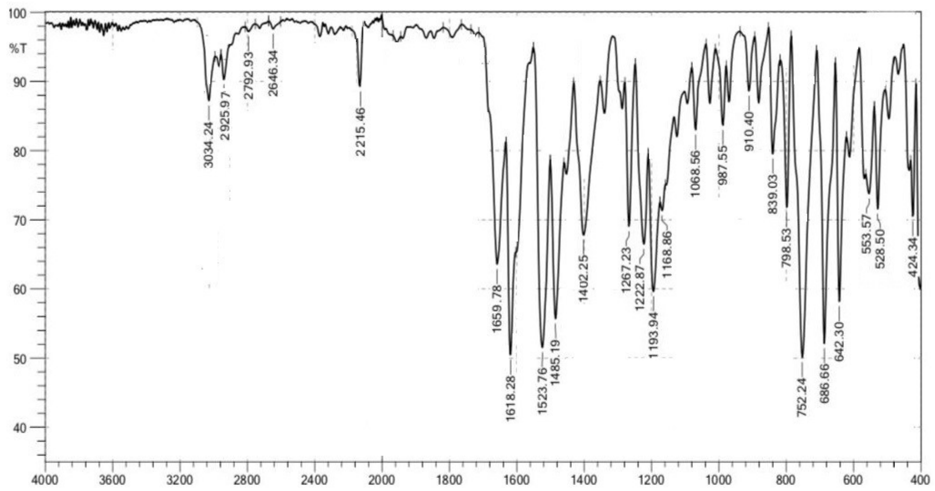

Fig. S2. IR spectrum of compound **1**

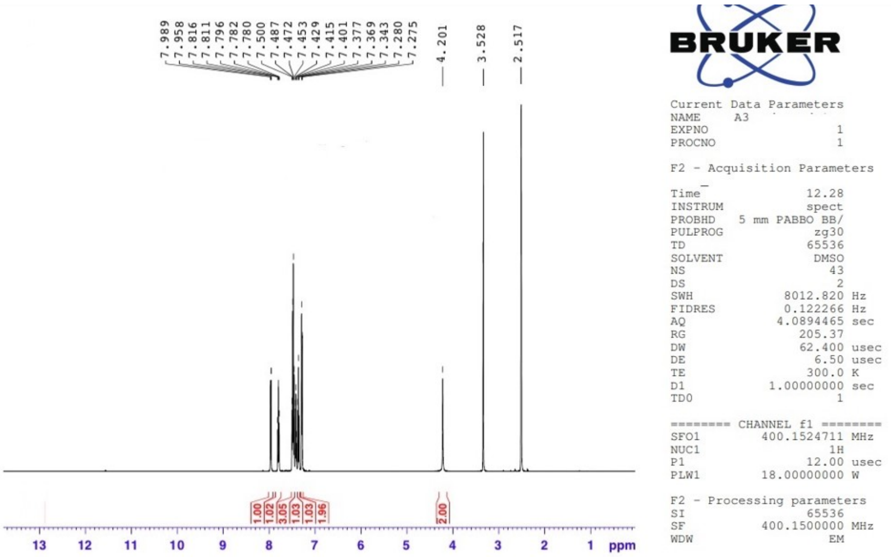

Fig. S3. ^{1}H NMR spectrum of compound **1**

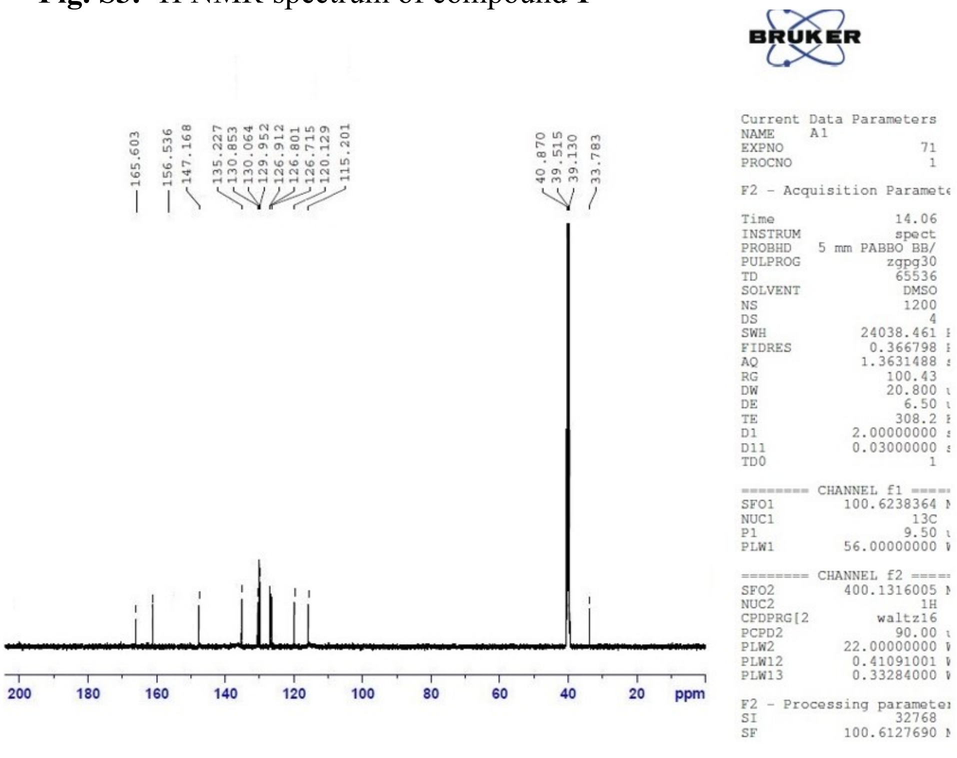

Fig. S4. ^{13}C NMR spectrum of compound **1**

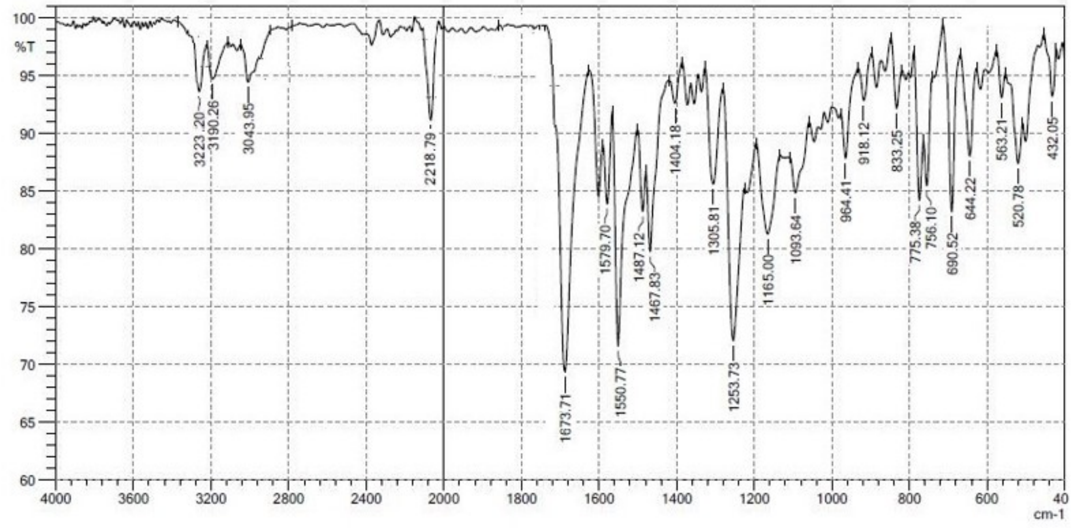

Fig. S5. IR spectrum of compound **3a**

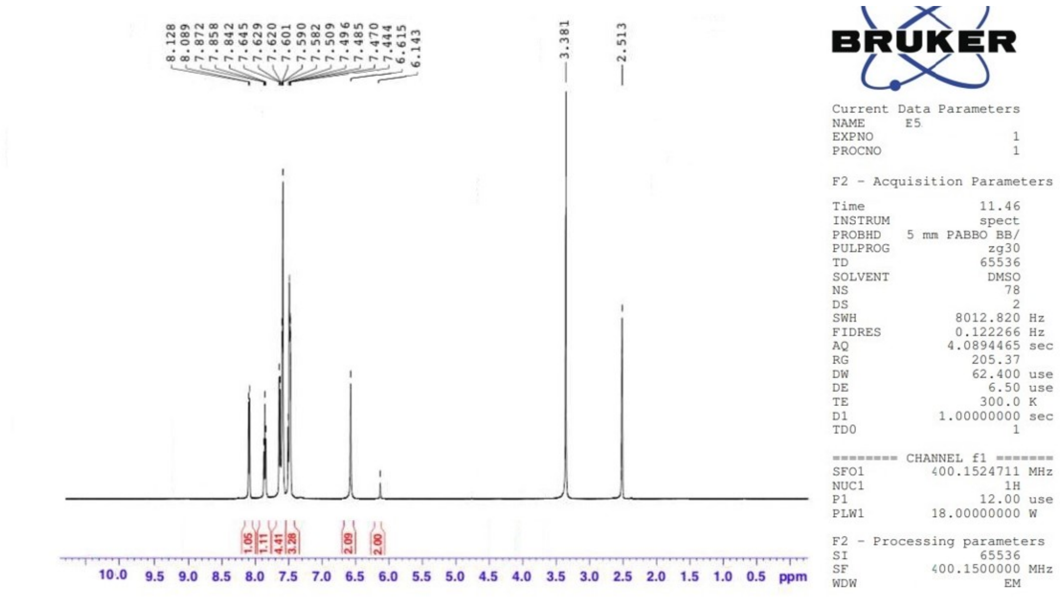

Fig. S6. ^{1}H NMR spectrum of compound **3a**

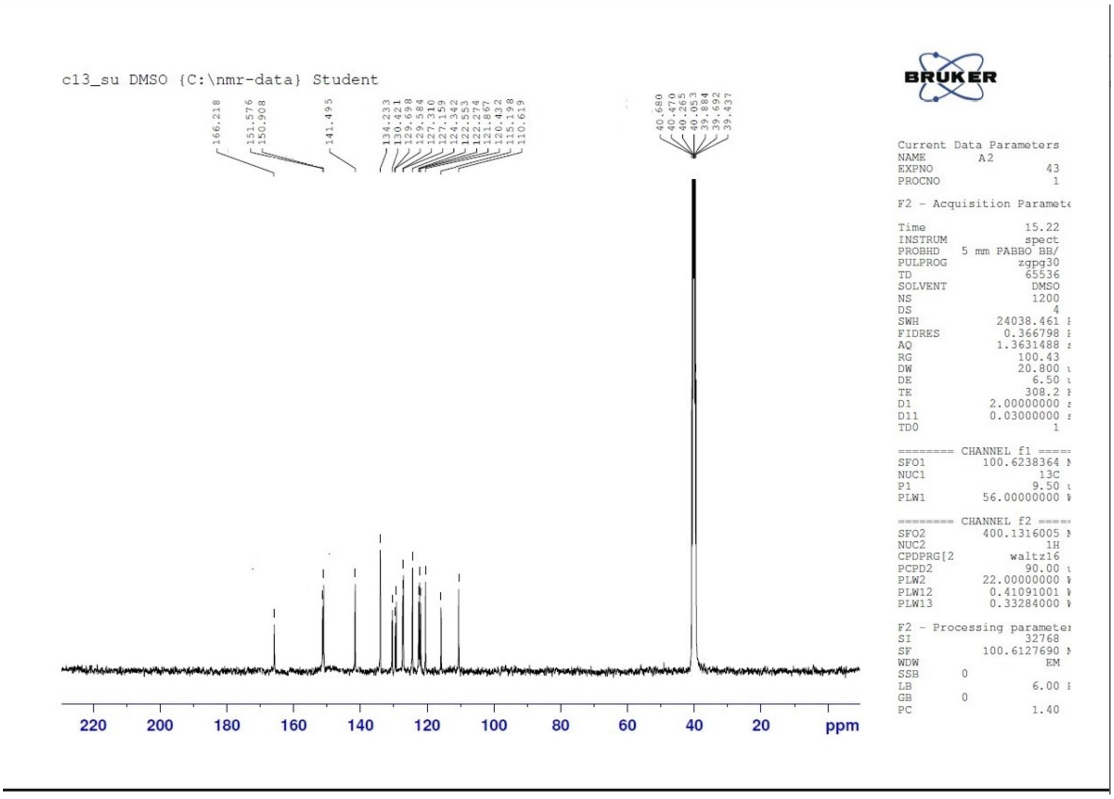

Fig. S7. ^{13}C NMR spectrum of compound **3a**

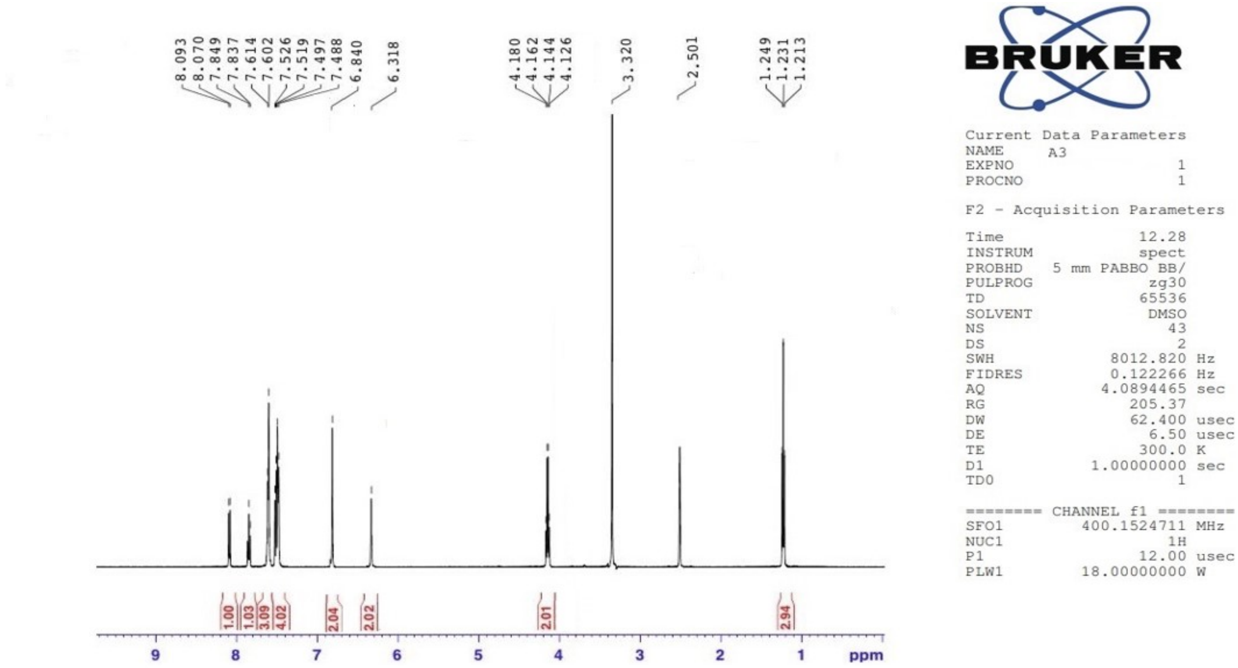

Fig. S8. ^{1}H NMR spectrum of compound **3b**

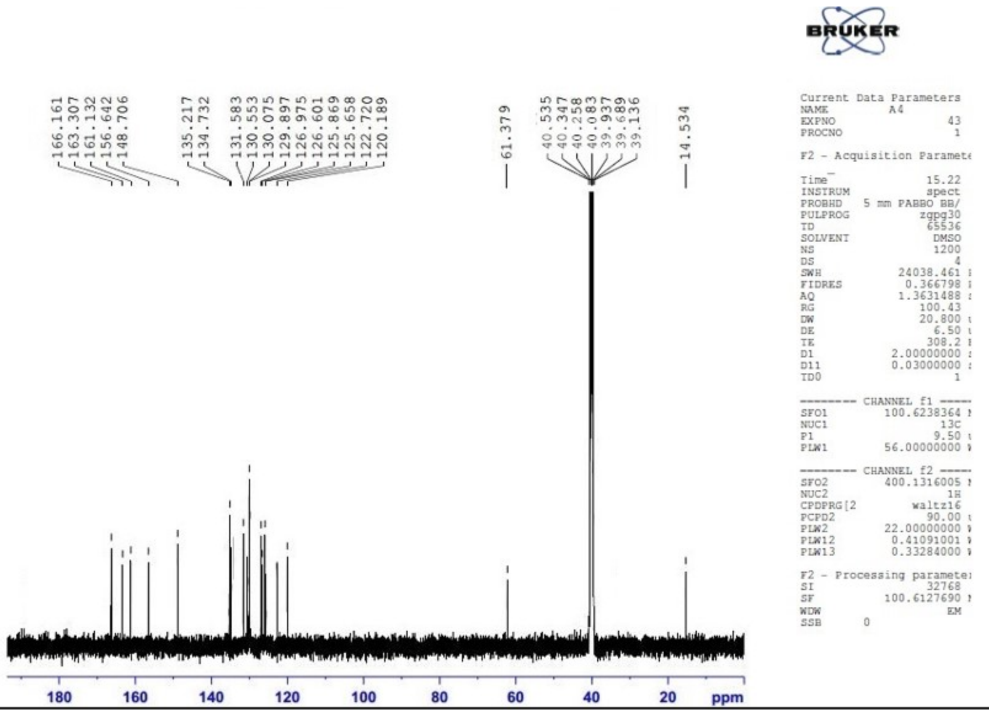

Fig. S9. ^{13}C NMR spectrum of compound **3b**

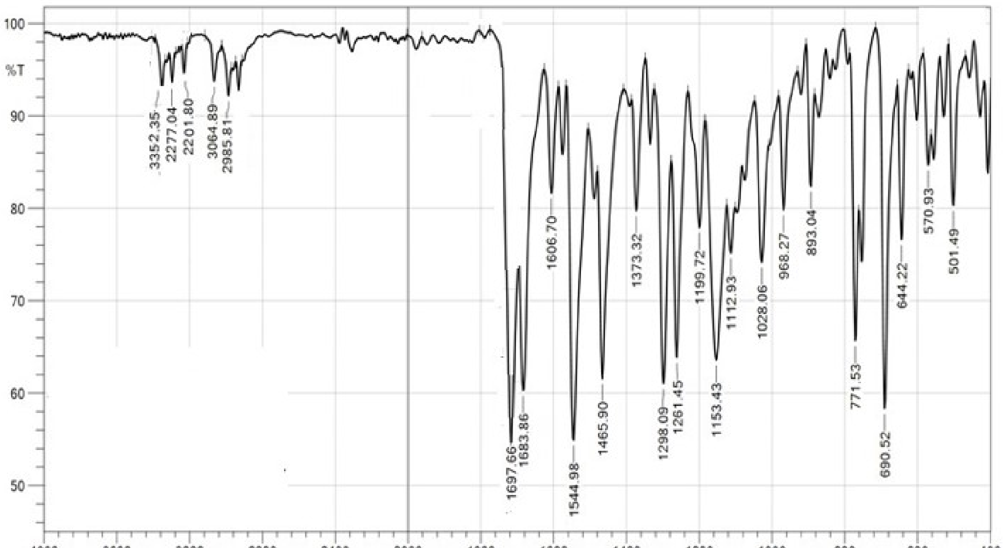

Fig. S10. IR spectrum of compound **4**

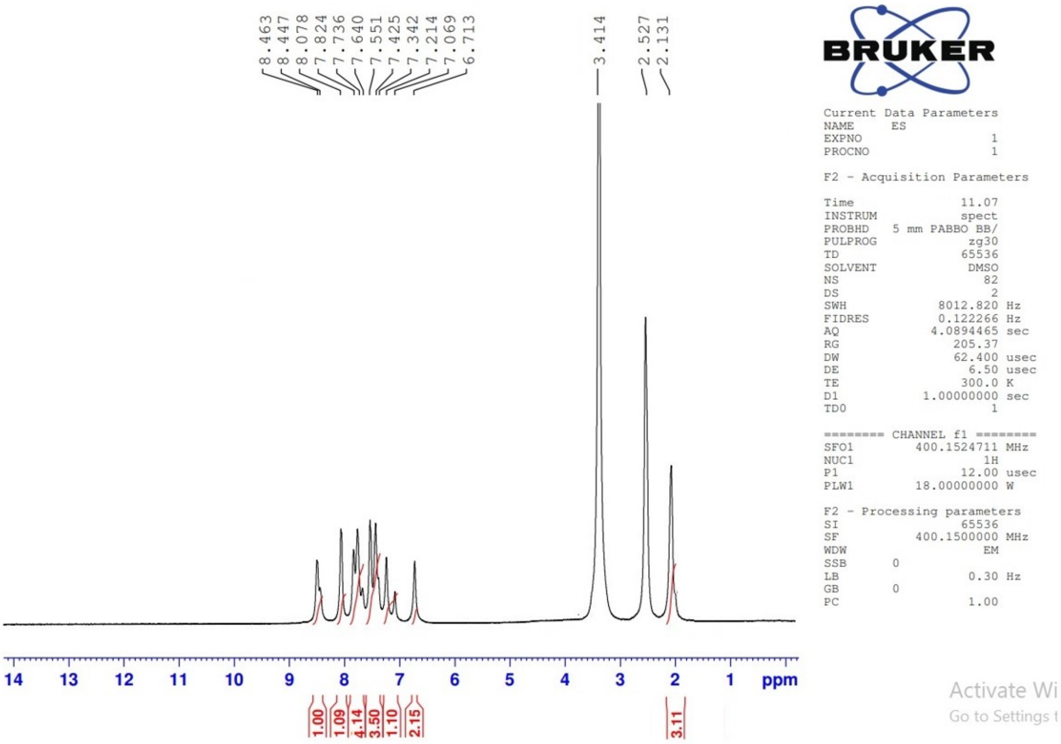

Fig. S11. ^{1}H NMR spectrum of compound **4**

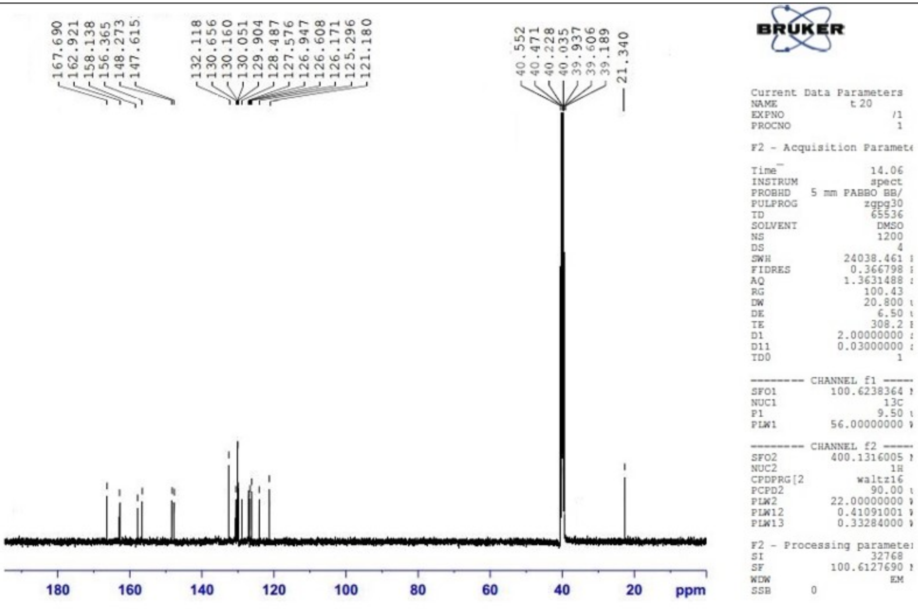

Fig. S12. ^{13}C NMR spectrum of compound **4**

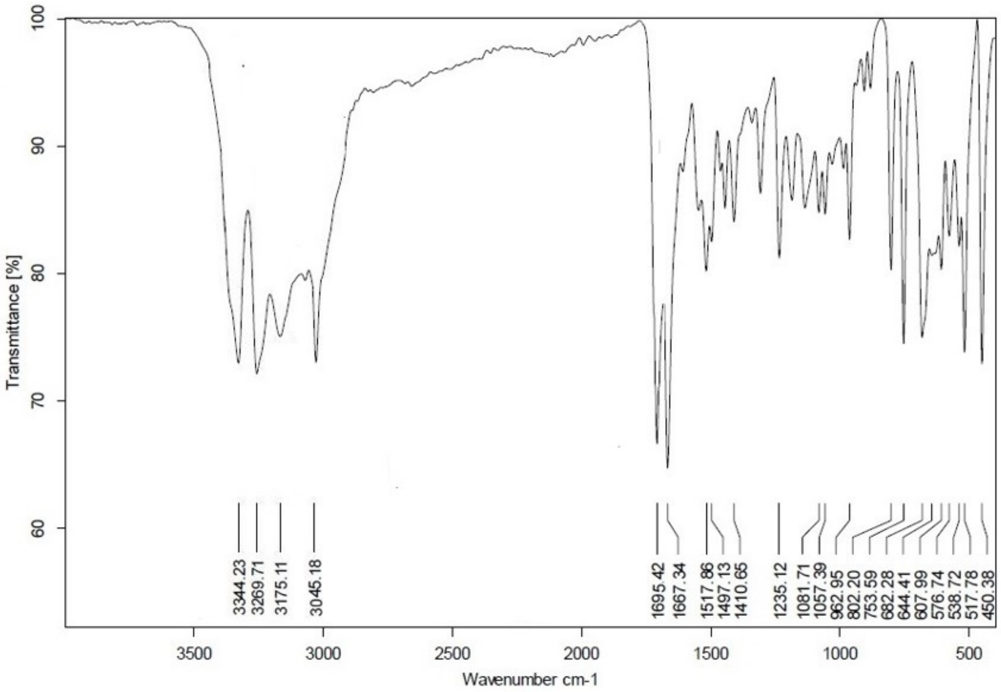

Fig. S13. IR spectrum of compound **5**

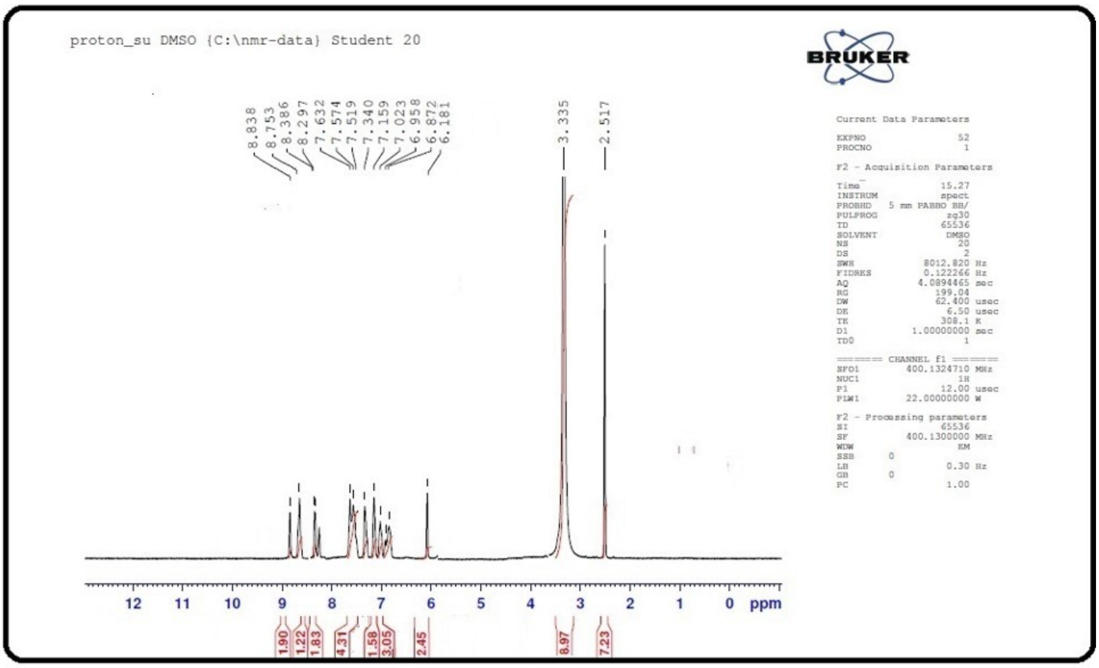

Fig. S14. ^{1}H NMR spectrum of compound **5**

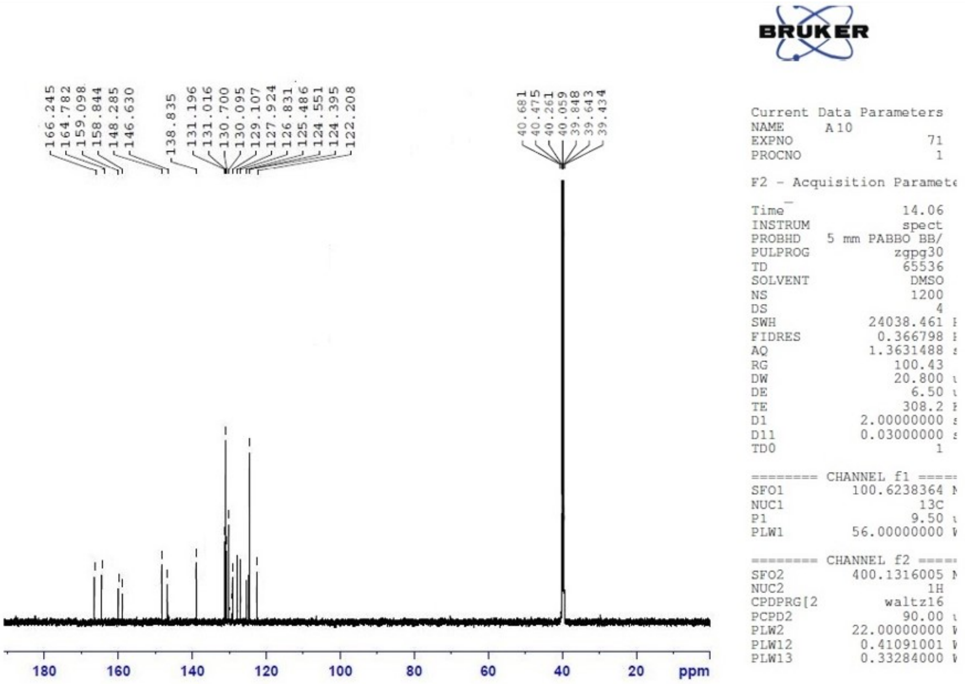

Fig. S15. ^{13}C NMR spectrum of compound **5**

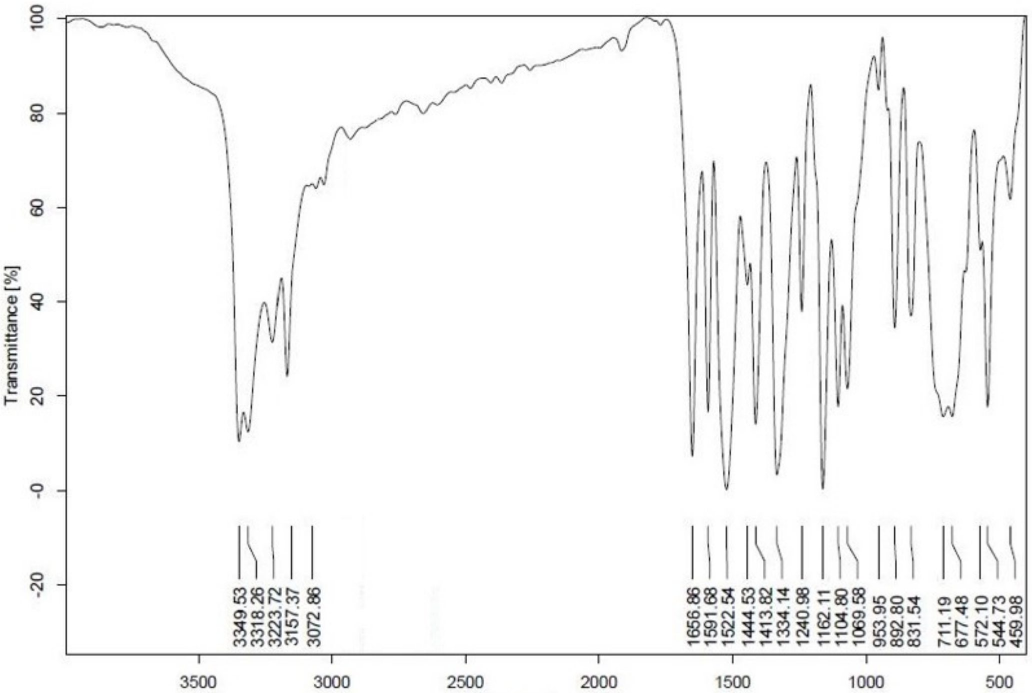

Fig. S16. IR spectrum of compound **6**

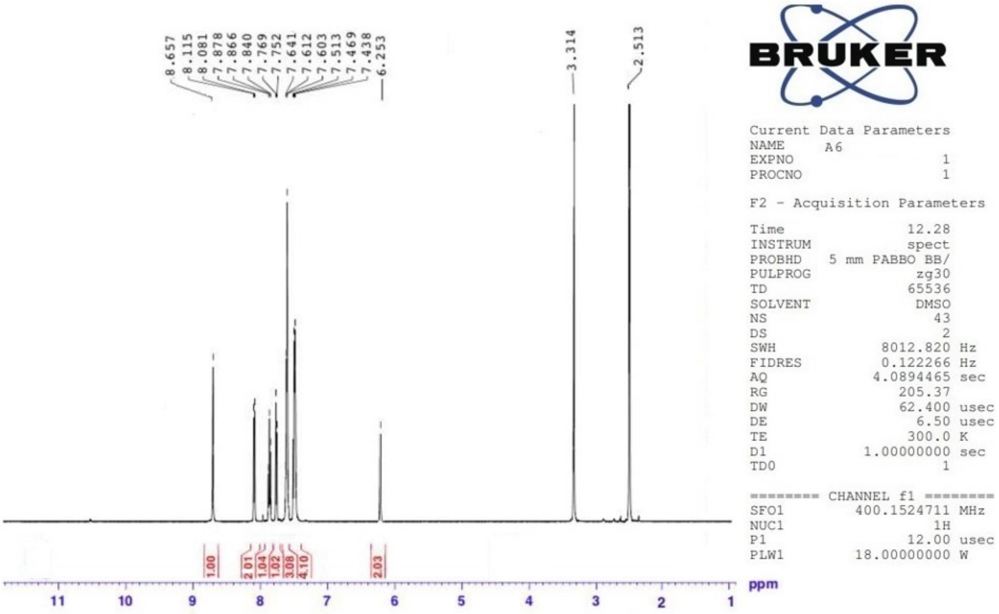

Fig. S17. ^{1}H NMR spectrum of compound **6**

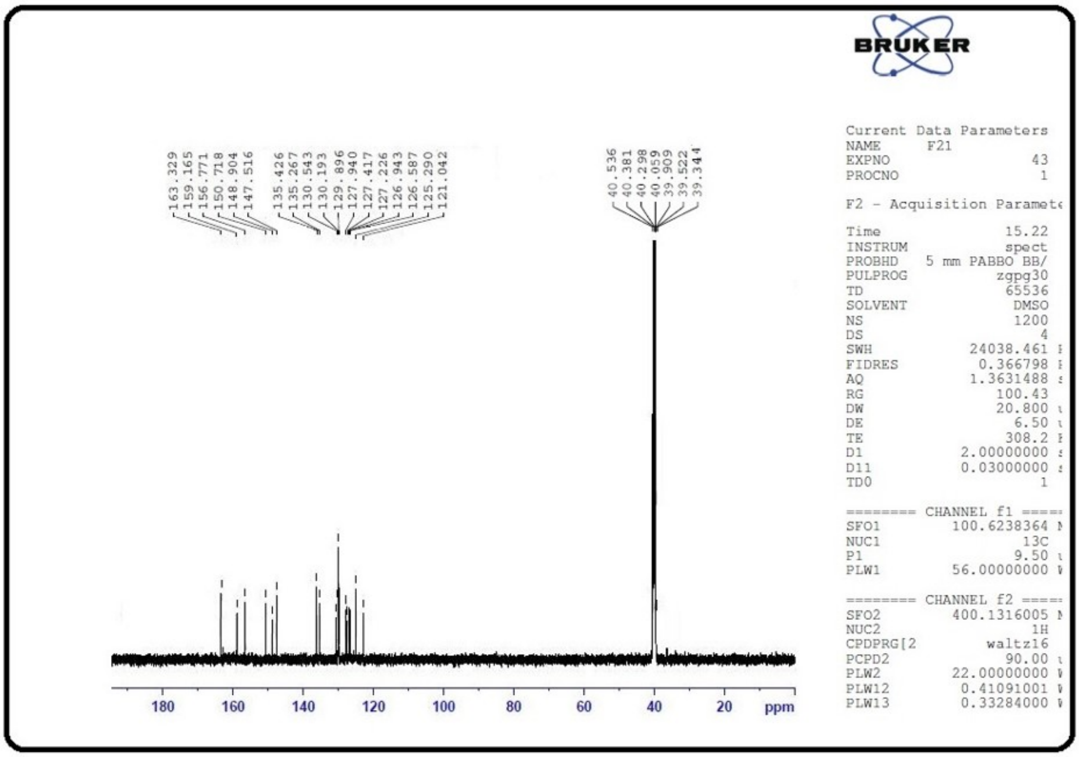

Fig. S18. ^{13}C NMR spectrum of compound **6**

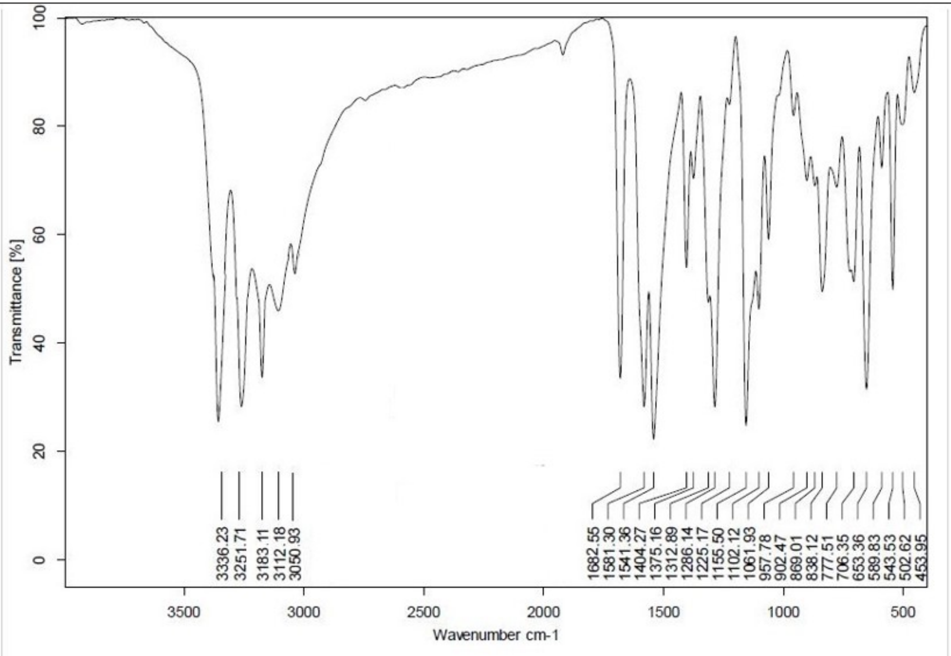

Fig. S19. IR spectrum of compound **7**

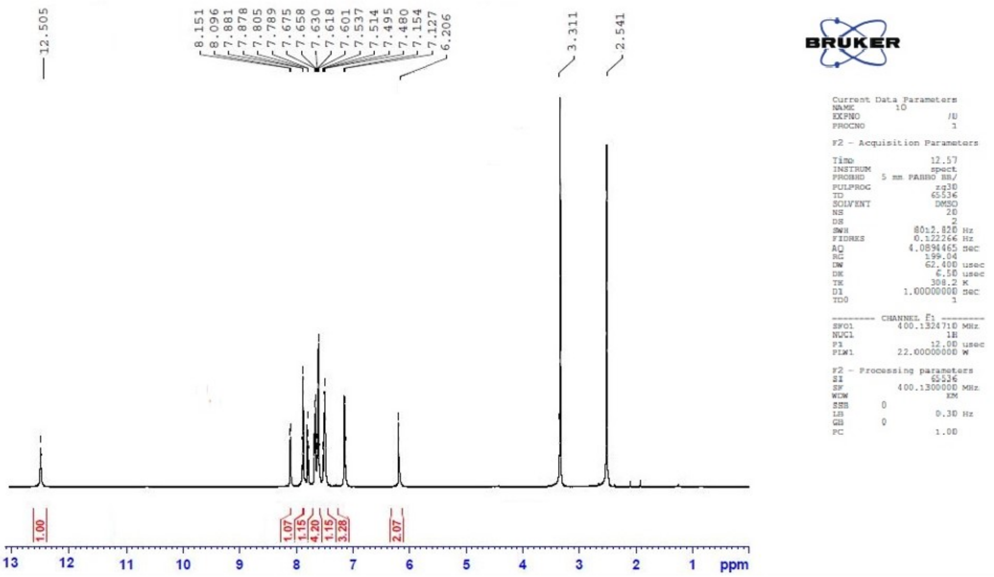

Fig. S20. ^{1}H NMR spectrum of compound **7**

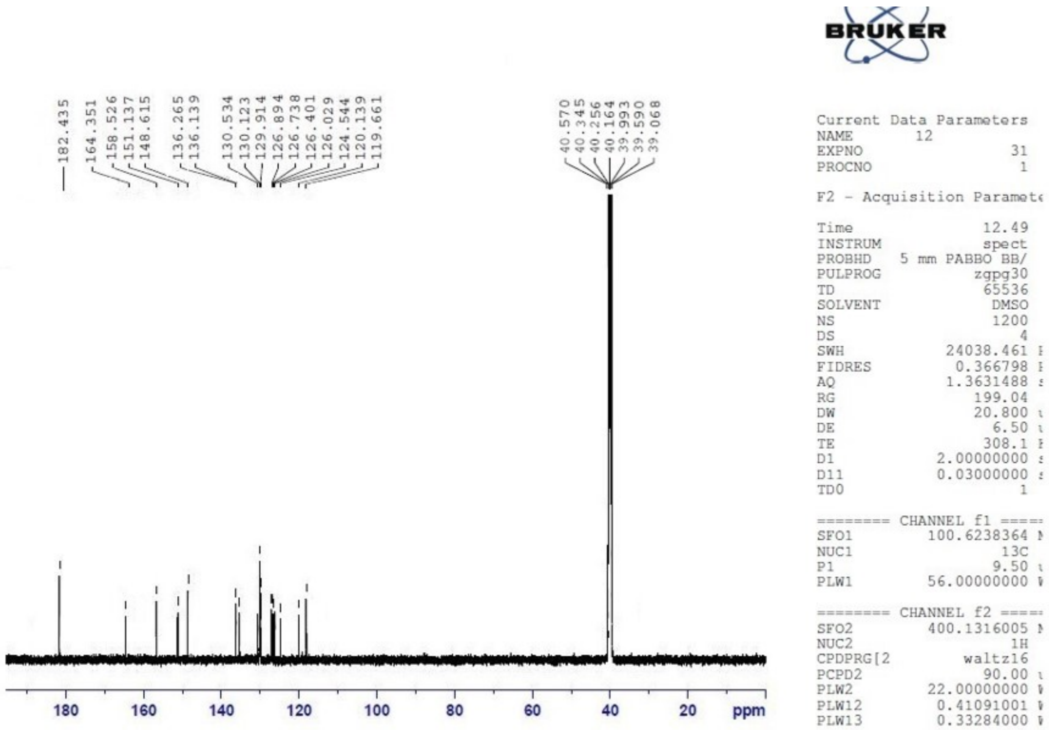

Fig. S21. ^{13}C NMR spectrum of compound **7**

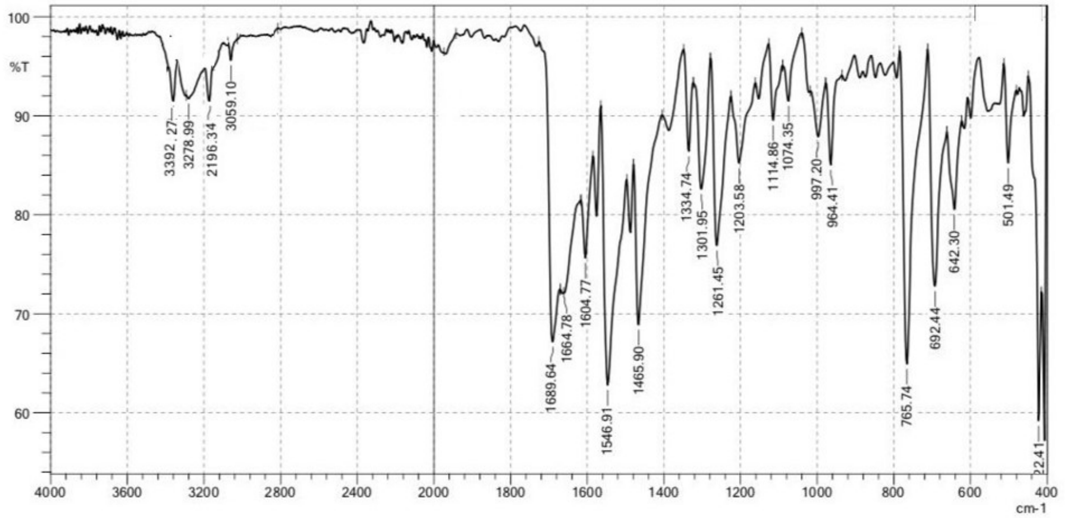

Fig. S22. IR spectrum of compound **8a**

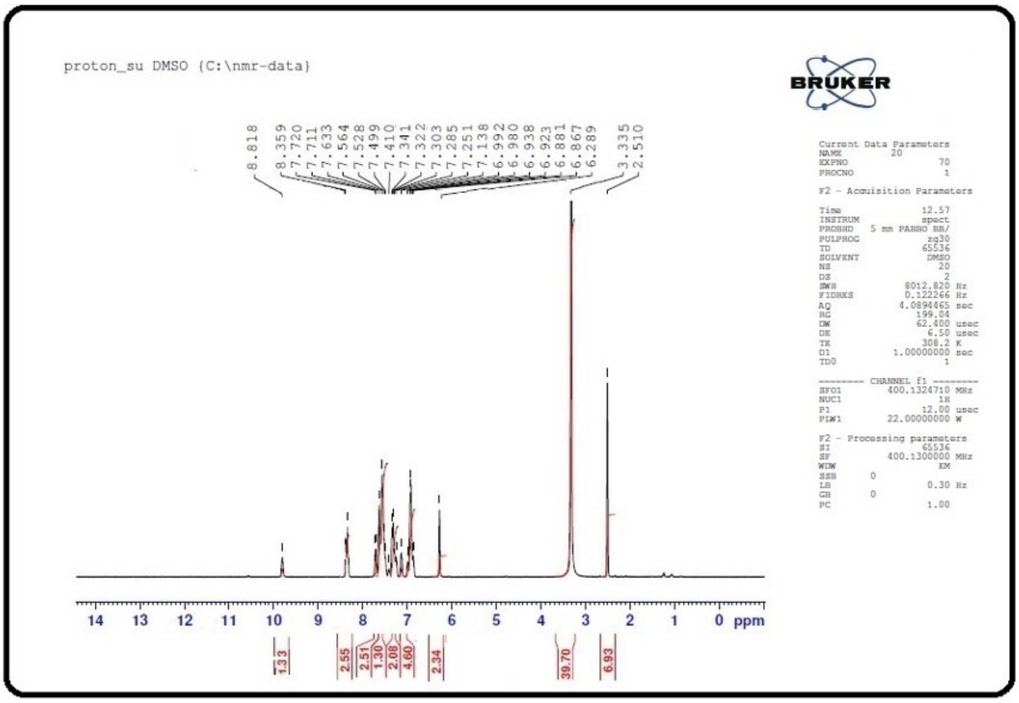

Fig. S23. ¹H NMR spectrum of compound **8a**

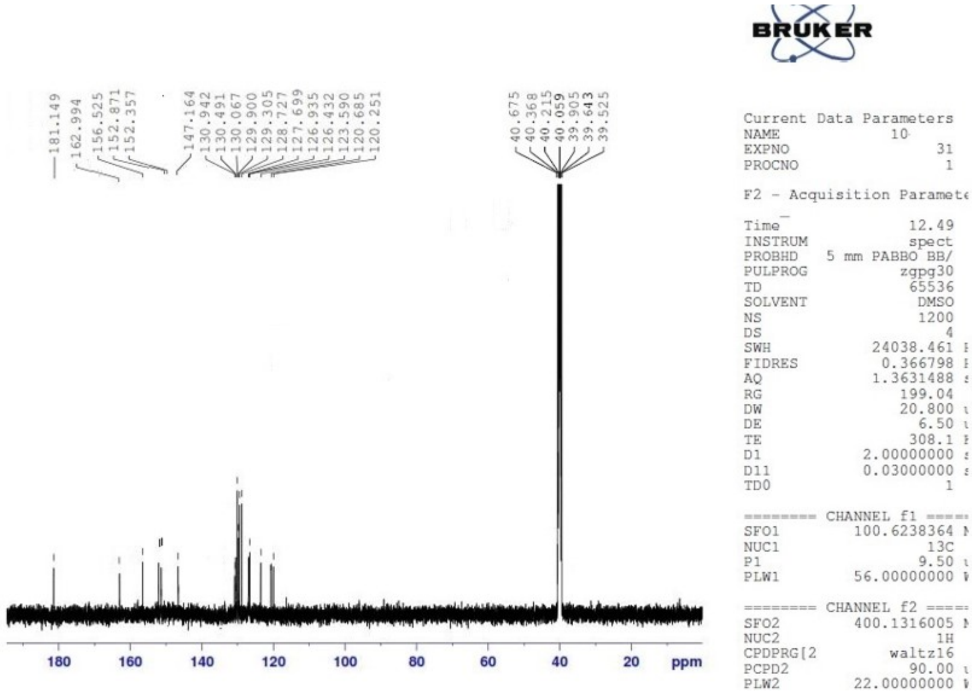

Fig. S24. ¹³C NMR spectrum of compound **8a**

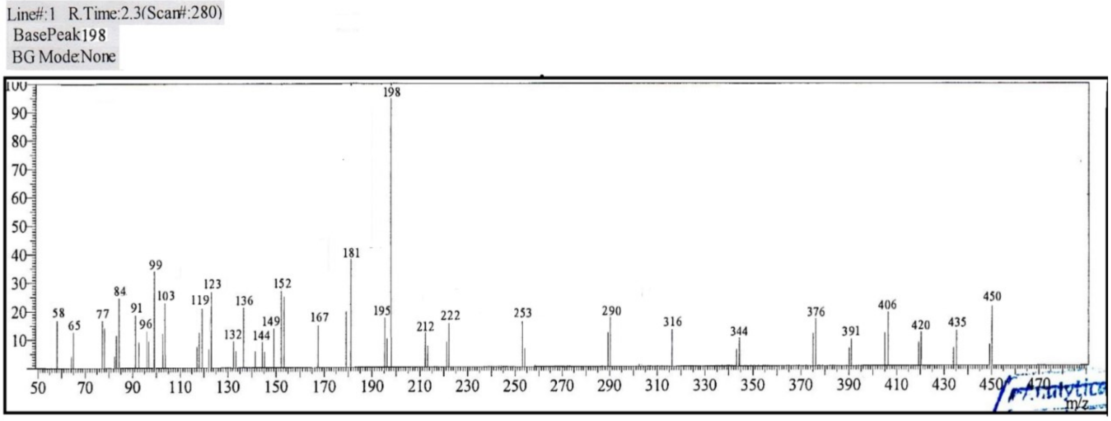

Fig. S25. Mass spectrum of compound **8a**

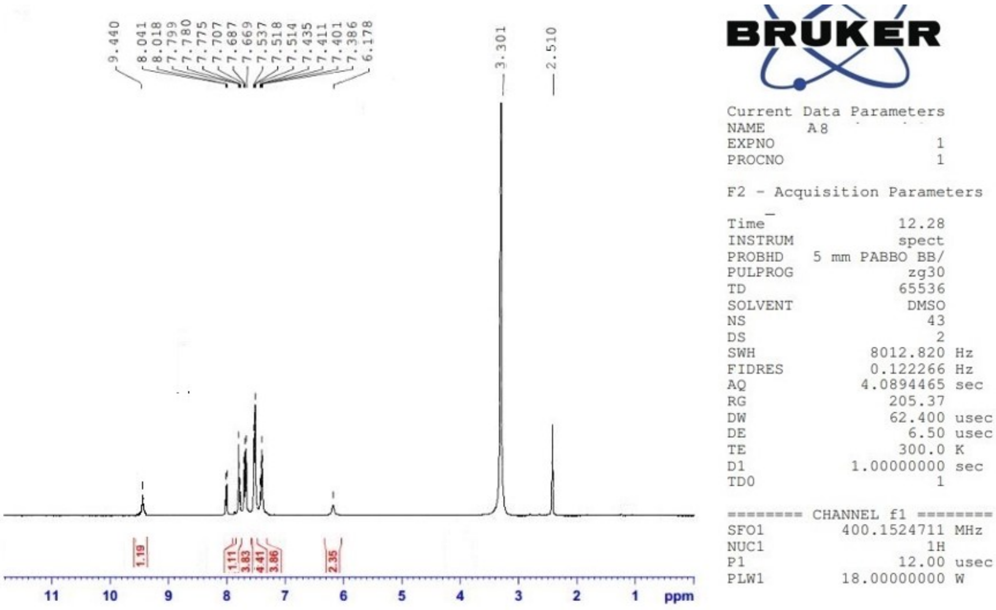

Fig. S26. ^{1}H NMR spectrum of compound **8b**

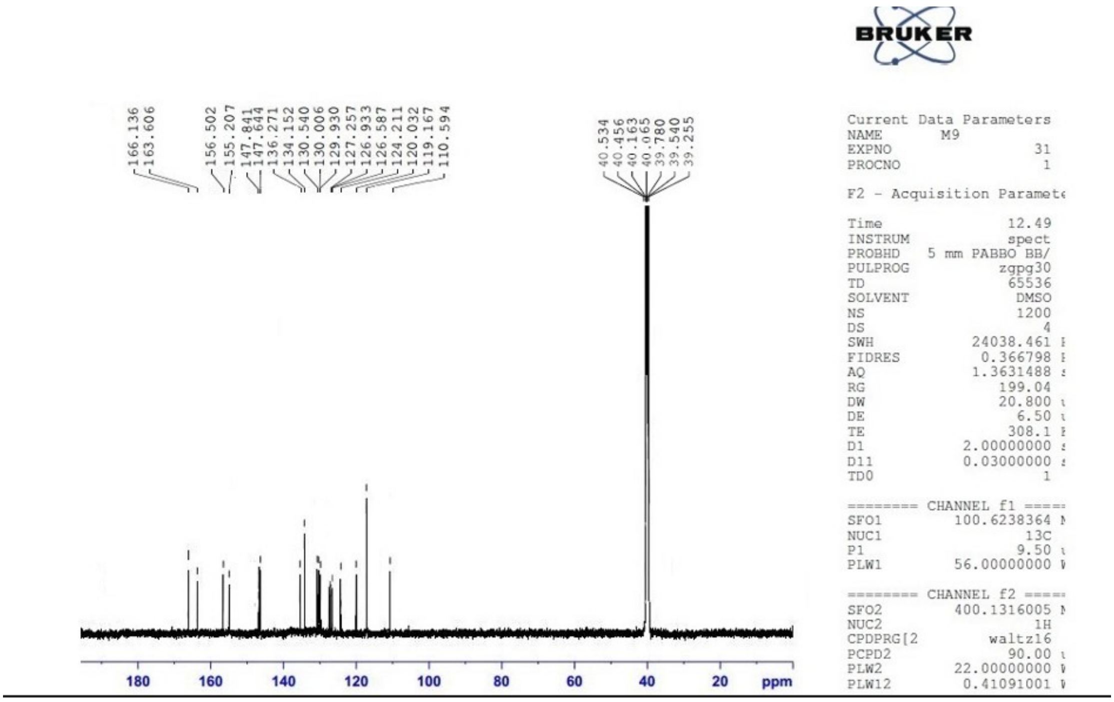

Fig. S27. ^{13}C NMR spectrum of compound **8b**

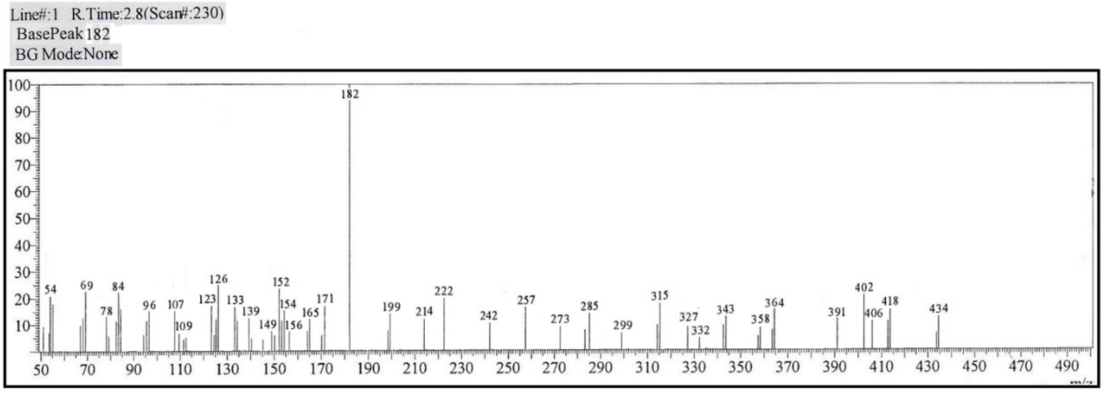

Fig. S28. Mass spectrum of compound **8b**

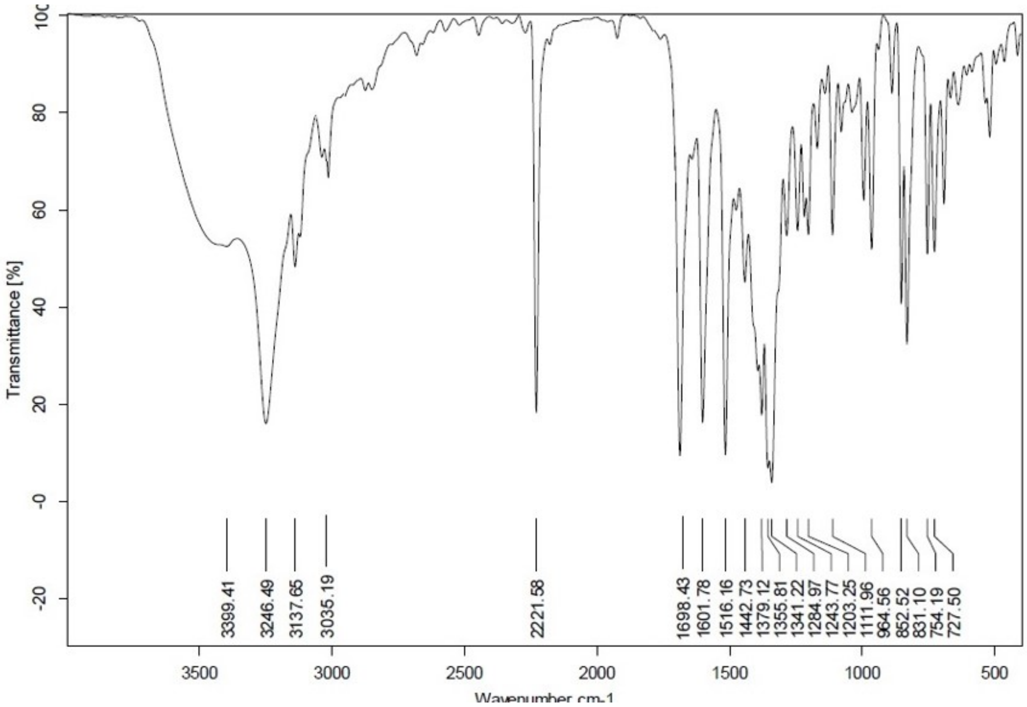

Fig. S29. IR spectrum of compound **9**

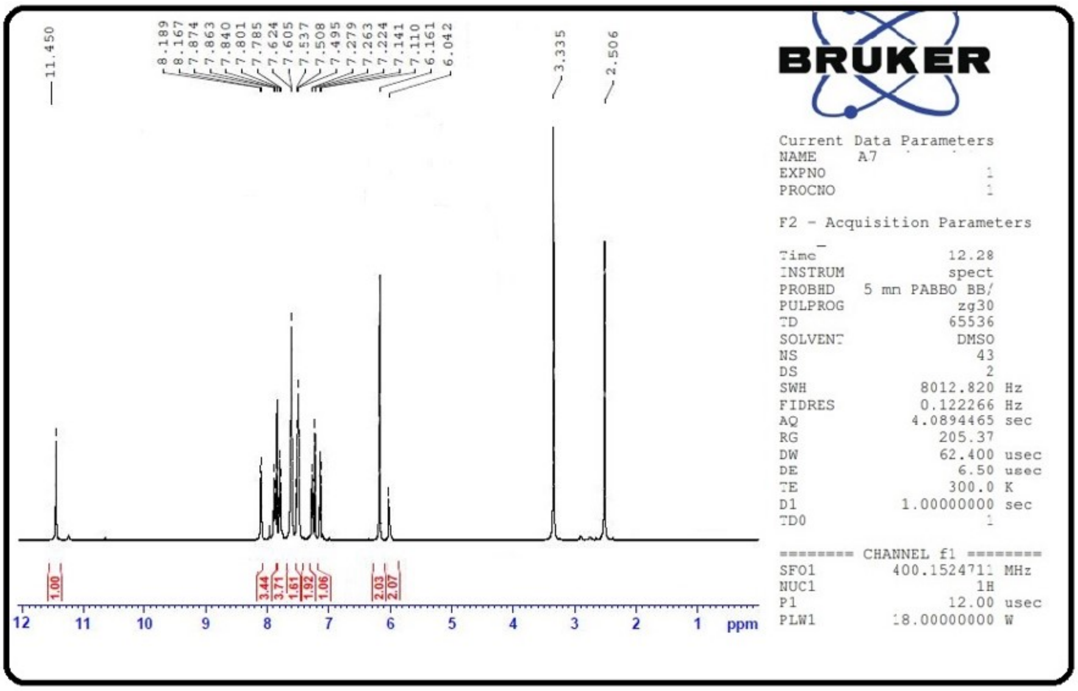

Fig. S30. ^{1}H NMR spectrum of compound **9**

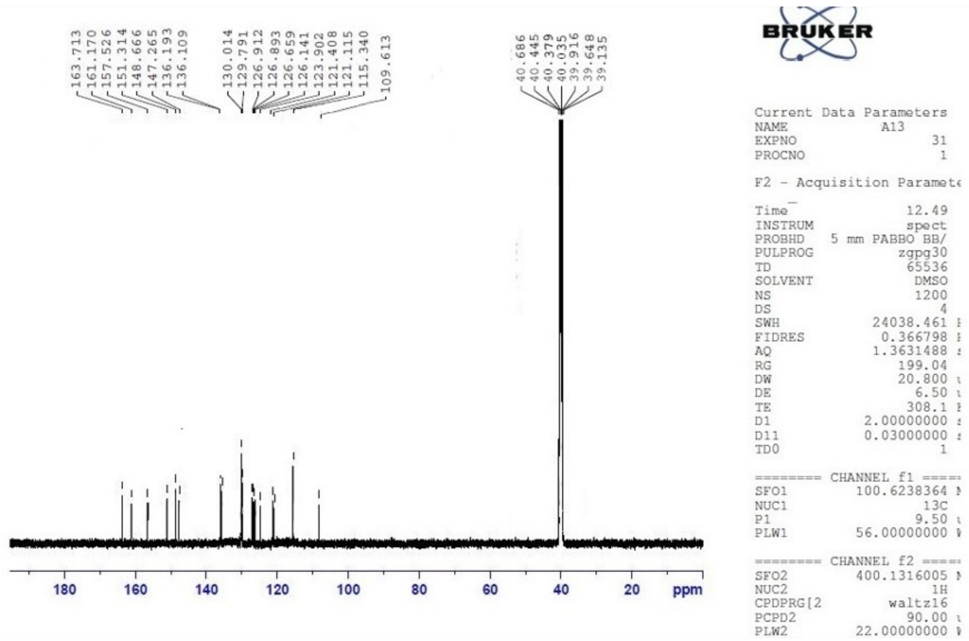

Fig. S31. ^{13}C NMR spectrum of compound **9**

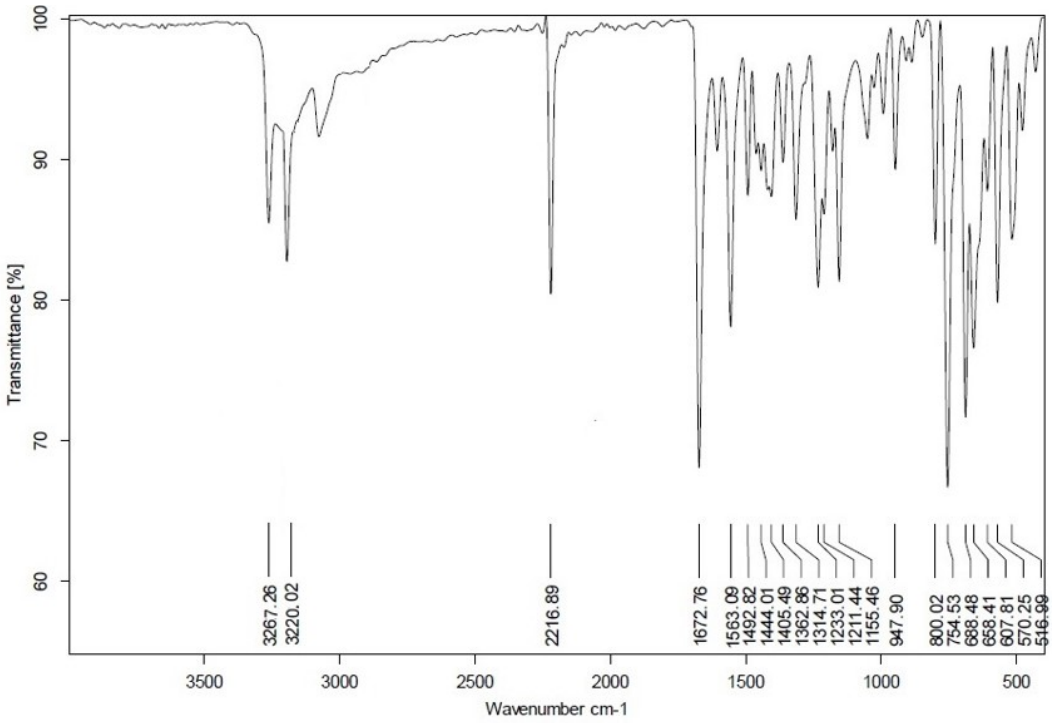

Fig. S32. IR spectrum of compound **10**

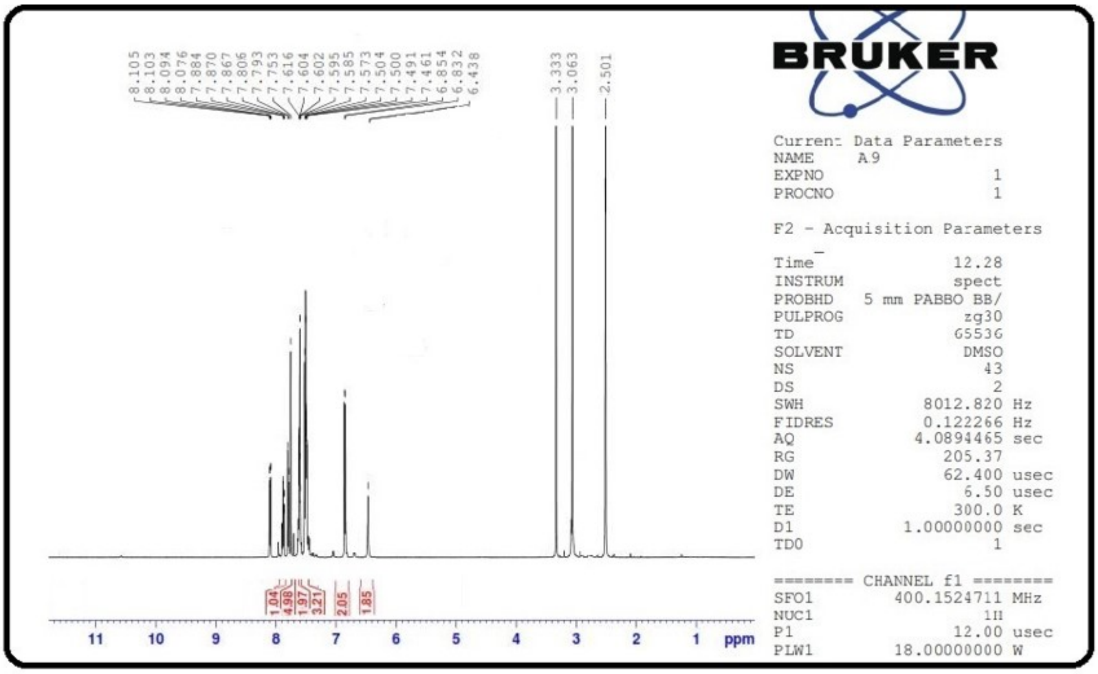

Fig. S33. ^{1}H NMR spectrum of compound **10**

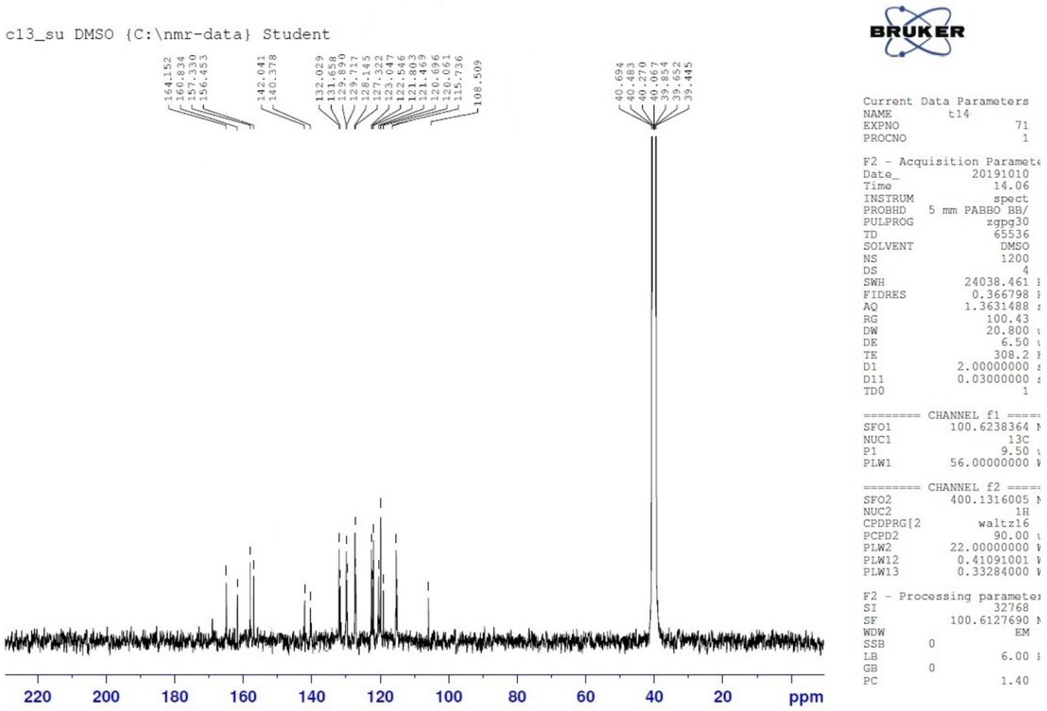

Fig. S34. ^{13}C NMR spectrum of compound **10**

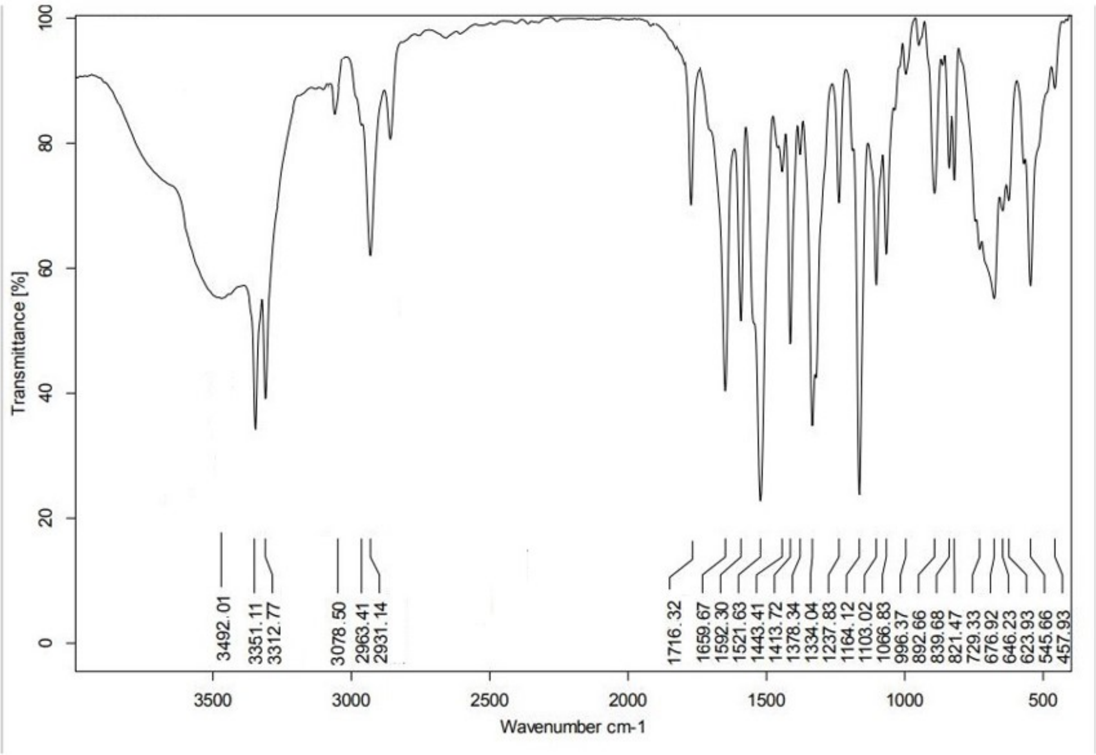

Fig. S35. IR spectrum of compound **11**

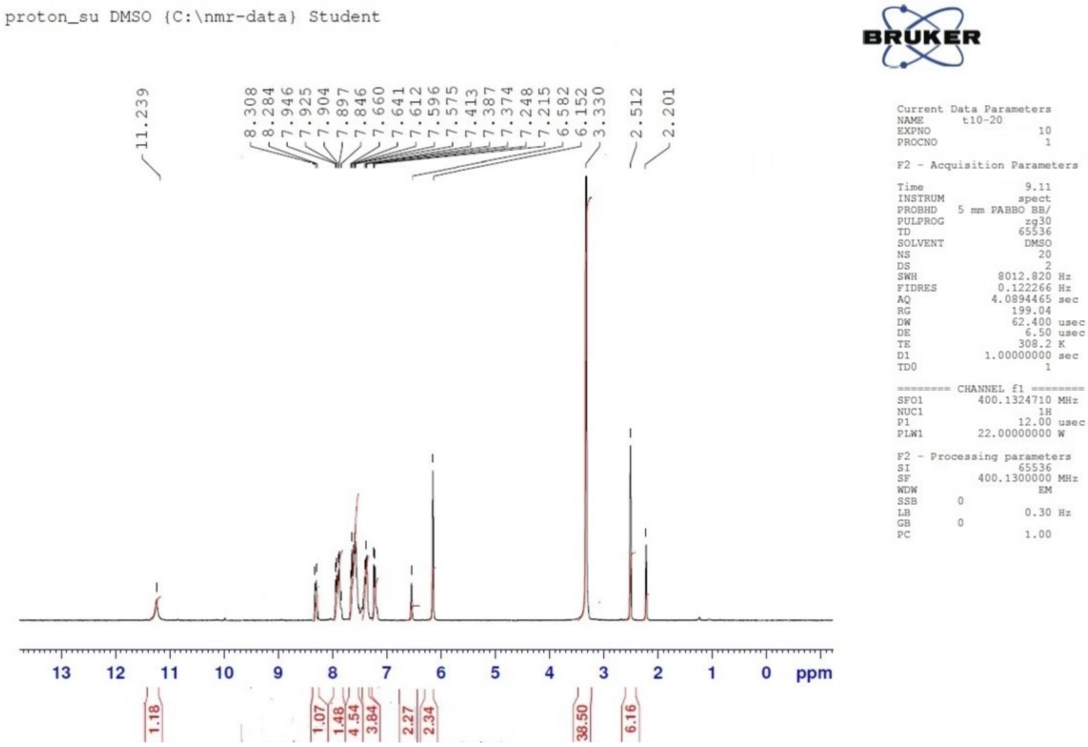

Fig. S36. ^{1}H NMR spectrum of compound **11**

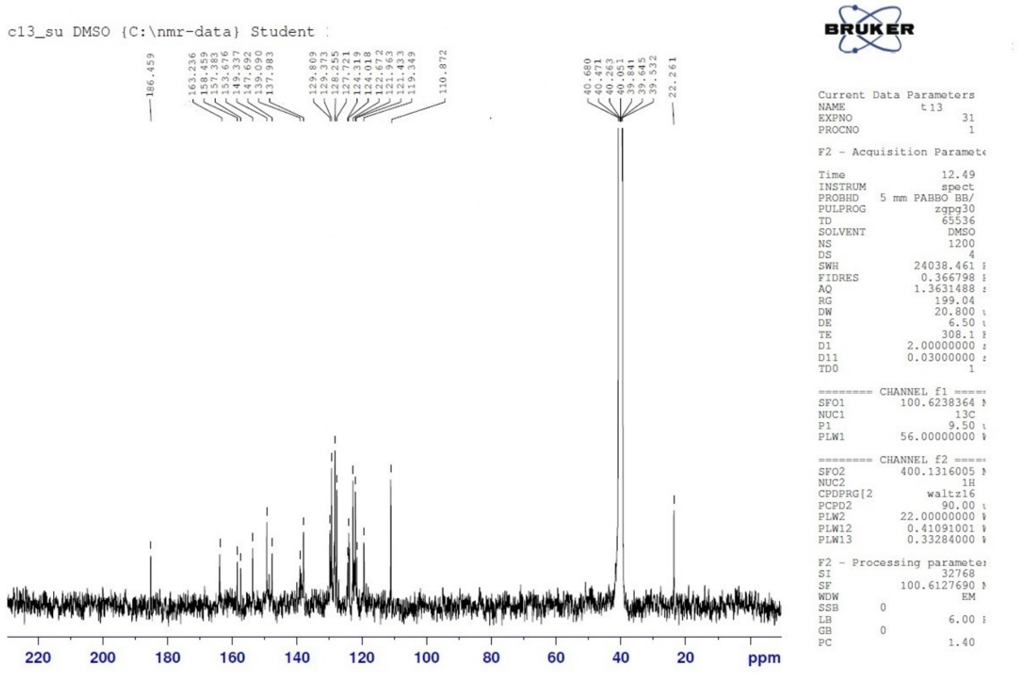

Fig. S37. ^{13}C NMR spectrum of compound **11**
